# Cost-effectiveness Analysis of Nirmatrelvir/Ritonavir Compared with Molnupiravir in Patients at High Risk for Progression to Severe COVID-19 in Japan

**DOI:** 10.36469/001c.129067

**Published:** 2025-02-24

**Authors:** Akira Yuasa, Hiroyuki Matsuda, Yosuke Fujii, Tendai Mugwagwa, Yuki Kado, Mizuki Yoshida, Koichi Murata, Yoshiaki Gu

**Affiliations:** 1 Japan Access & Value Pfizer Japan Inc.; 2 Real World Evidence Solutions & HEOR IQVIA Solutions Japan G.K; 3 Pfizer R&D Japan; 4 Global Access & Value Pfizer Inc.; 5 Real World Evidence Solutions & HEOR IQVIA Solutions Japan G.K.; 6 Medical Affairs Pfizer Japan Inc.; 7 Department of Infectious Diseases Institute of Science Tokyo

**Keywords:** nirmatrelvir, molnupiravir, cost-effectiveness analysis, COVID-19, Japan

## Abstract

**Background:** Nirmatrelvir/ritonavir (NMV/r) and molnupiravir are oral antiviral drugs approved for the treatment of early symptomatic patients with mild to moderate COVID-19 at high risk of progression to severe disease in Japan. **Objective:** This study evaluated, from a Japanese payer perspective, the cost-effectiveness of NMV/r compared with molnupiravir among patients with COVID-19. **Methods:** This cost-effectiveness model describes the COVID-19 disease history and the impact of antiviral treatment on short-term and long-term outcomes. Nirmatrelvir/ritonavir was compared with molnupiravir, and in the scenario analysis NMV/r was compared with standard of care over a lifetime horizon. **Results:** When compared with molnupiravir, NMV/r showed higher quality-adjusted life years (QALYs) (15.752 vs 15.739) and higher total cost (¥6 248 014 vs ¥6 245 829 [US 44 136.86vs44 121.42]). The incremental cost-effectiveness ratio was ¥164 934 (US 1165.12)perQALYgained,whichwaslowerthanthewillingness−to−pay(WTP)thresholdinJapan(¥5 000 000/QALY[US35 320.71/QALY]). In the scenario analysis, the incremental cost-effectiveness ratio was ¥3 646 821 (US 25 761.66)perQALYgained.One−waysensitivityanalysisandprobabilisticsensitivityanalysisshowedthatNMV/rwascost−effectivecomparedwithmolnupiravirandstandardofcareconsistently.Allresultsofone−waysensitivityanalysisshowedthattheincrementalcost−effectivenessratioswerebelowtheWTPthresholdinJapan.Theresultofthecost−effectivenessacceptabilitycurveshowedthattheprobabilitythattheincrementalcost−effectivenessratiowasbelowtheWTPof¥5 000 000/QALY(US35 320.71/QALY) was 100.00%. **Conclusion:** Nirmatrelvir/ritonavir is cost-effective compared with molnupiravir and standard of care from a Japanese payer perspective. This study provides evidence for the cost-effectiveness of NMV/r for patients with COVID-19.

## BACKGROUND

Coronavirus disease 2019 (COVID-19) caused by severe acute respiratory syndrome coronavirus 2 (SARS-CoV-2) became a pandemic in 2020, overwhelming healthcare systems and draining financial reserves all around the world. Omicron, a new variant that emerged in 2021, was more transmissible and rapidly spread to every country in the world. It quickly became the dominant variant, replacing preceding ones. In Japan, Omicron was circulating 4.3 times more than the preceding variant, infecting 7.3 million people, compared with 1.7 million reported in the previous 2 years.^1^ The Japanese Clinical Practice Guidelines for the Management of Sepsis and Septic Shock 2020 developed the Japanese rapid/living recommendations on drug management for COVID-19. The first edition of this clinical practice guideline was released in 2020 and has been revised periodically with updated evidence.^2^ In Japan, the Omicron variant began to be replaced by other variants in early 2022, resulting in sudden changes in antiviral drugs and their status.

During the early stages of the pandemic, different antiviral drugs, immunosuppressive drugs, anticoagulants, and monoclonal antibody therapies were used as early treatments to prevent disease progression. In Japan, the oral antiviral agents nirmatrelvir/ritonavir (NMV/r), and molnupiravir are recommended for the treatment of adult patients with mild to moderate COVID-19 at high risk of progression to severe COVID-19 as outpatient treatments for COVID-19.^3^

Nirmatrelvir/ritonavir, a combination drug comprising a protease inhibitor and a CYP3A inhibitor (pharmacokinetic enhancer), was evaluated for its safety and efficacy among non-hospitalized adults with mild to moderate COVID-19 at high risk for progression to severe disease in the phase II/III Evaluation of Protease Inhibitor for COVID-19 in High-Risk Patients (EPIC-HR) trial.^4^ Nirmatrelvir/ritonavir showed a relative risk reduction in COVID-19–related hospitalization or death from any cause through day 28 by 87.8% compared with placebo in patients treated within 5 days after symptom onset.

Molnupiravir, a ribonucleoside analog, achieved a 30% reduction in the risk of hospitalization or death in at-risk, unvaccinated adults with COVID-19 in the phase III MOVe-OUT trial. Besides clinical trials, real-world studies have also demonstrated the effectiveness of NMV/r and molnupiravir in reducing hospitalization or death in COVID-19 patients.^5–7^

Cost-effectiveness analysis (CEA) of NMV/r have been reported in several studies.^8–11^ In 2020, the Institute of Clinical and Economic Review in the United States conducted a special assessment on clinical and economic outcomes of NMV/r and molnupiravir for mild to moderate COVID-19 among outpatients with a high risk of progression to severe disease. In the evidence report issued by the Institute of Clinical and Economic Review, the cost-effectiveness of each treatment was estimated using estimates of relative treatment effectiveness from their pivotal trials.^12^ The evidence reports also reported the health-benefit price benchmarks of NMV/r and molnupiravir from a healthcare sector perspective. A recent CEA study using a similar model conducted by Institute of Clinical and Economic Review reported that NMV/r was cost-effective compared with best supportive care from a US healthcare perspective.^13^ However, CEA results of NMV/r compared with molnupiravir have not been published, including in Japan.

At the time of analysis, molnupiravir was launched prior to NMV/r in Japan, where it is the most commonly prescribed oral anti-COVID-19 drug. In Japan, public funding for anti-COVID-19 drugs has ended since April 2024; therefore, a cost-effective drug needs to be identified. Assessing whether NMV/r or molnupiravir is more cost-effective is highly significant for clinical practice in Japan. Additionally, as public funding for COVID-19 treatment in Japan has ended, the cost-effectiveness of anti-COVID-19 drugs relative to standard of care (SoC) could provide important evidence. We conducted a study to compare the cost-effectiveness of NMV/r with molnupiravir in response to the motivation to assess the cost-effectiveness of anti-COVID-19 drugs.

## METHODS

### Model Approach

An Excel-based CEA model was adapted to the Japanese setting based on the methodology described by Carlson et al.^13^ The Japanese model describes the short-term acute infection period using a decision tree followed by a lifetime Markov model to capture the long-term impact of COVID-19 infection. The model was used to compare the cost-effectiveness of NMV/r treatment compared with molnupiravir treatment of patients with mild to moderate COVID-19 with risk factors for severe COVID-19 from a Japanese payer perspective. The model captures associated costs and health benefits in terms of quality-adjusted life years (QALYs), which are discounted at 2% for both costs and benefits as per Japanese health technology assessment (HTA) guidelines. Results are reported as total costs, incremental costs, total QALYs, incremental QALYs and incremental cost-effectiveness ratios (ICERs). The ICER was compared with a Japanese willingness-to-pay (WTP) threshold of ¥5 000 000 per QALY (US $35 320.71/QALY). All prices were stated in Japanese yen at a conversion rate of US $1 = ¥141.56.^14^

### Model Structure

**Figure 1.**
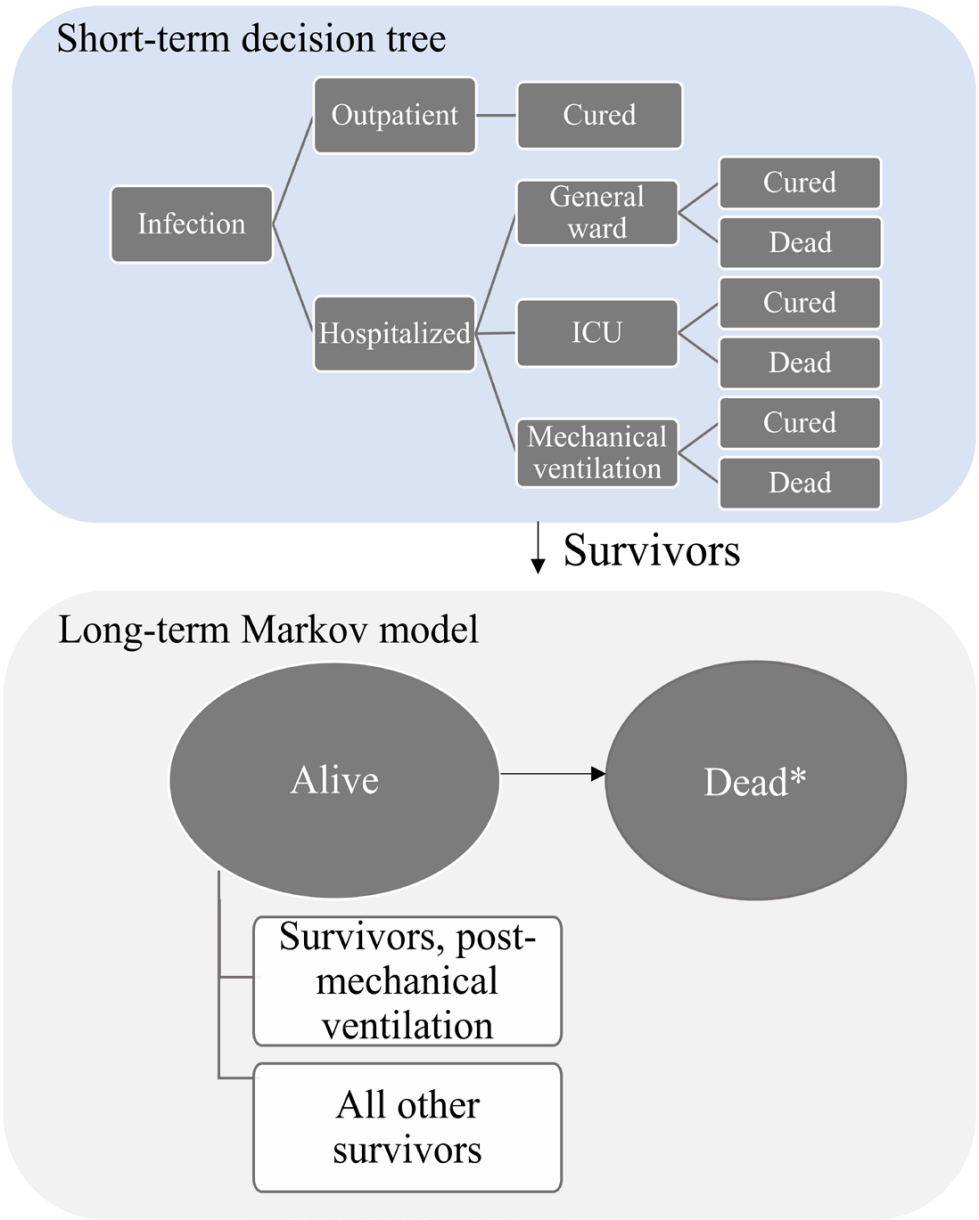
Hybrid Cost-Utility Model Structure, Including Decision Tree and Lifetime Markov Model Abbreviation: ICU, intensive care unit. *Death due to general population mortality.

A hybrid cost-utility model captures the short-term acute infection period using a decision tree followed by a lifetime Markov model to capture the long-term impact of COVID-19 infection (**Figure 1**). The model therefore considered the lifetime module (100-year time horizon) to capture the potential long-term sequelae of COVID-19 and the number of deaths to capture the impact on life expectancy and QALYs, according to Japanese HTA guidelines.^15^ The model used a closed cohort approach with 1000 symptomatic high-risk patients infected with COVID-19 to progress to severe COVID-19. The risk factors included age of at least 60 years, hypertension, diabetes mellitus, chronic kidney disease, and chronic lung disease.^16^ Initially, all patients were in an outpatient setting, where they might recover or progress to severe COVID-19 resulting in hospital admission. The model accounts for healthcare resource use (HCRU) in the outpatient setting (eg, practitioners office, emergency room, home health service) and in the hospital setting (eg, general ward, intensive care unit [ICU] admission with and without mechanical ventilation). All patients may develop post-acute COVID-19 syndrome (PACS), but only those hospitalized for COVID-19 may die.

The short-term decision tree spans a 1-year period at a daily cycle length and evaluates the consequences of daily SARS-CoV-2 infections alongside inpatient and outpatient care. Decision trees have been used previously in studies evaluating health economic outcomes for respiratory illnesses.^17,18^ Survivors of the short-term module enter the lifetime module, consisting of a 2-state Markov model (alive and dead) run on an annual cycle length. For individuals who survive after COVID-19 (alive), the transition probabilities related to mortality are considered, and the general mortalities of the Japanese population life table are applied.^19^ Individuals in the alive state are stratified by whether they experience long-term sequelae of COVID-19.

### Model Inputs

**Intervention and comparators:** The intervention used in this study was NMV/r; molnupiravir was the comparator. In the scenario analysis, SoC was considered the comparator in place of molnupiravir. The costs and effects of treatment, which serve as trade-offs between NMV/r and SoC, are allocated to each arm based on the specific intervention or comparator being considered.

**Patient population**: Adult nonhospitalized symptomatic SARS-CoV-2–infected patients at risk of progressing to severe COVID-19 illness were included in this analysis. The definition of risk factors for severe illness, based on a U.S. Centers for Disease Control and Prevention report and Hammond et al, includes age 60 years and older, hypertension, diabetes mellitus, chronic kidney disease, and chronic lung disease.^4,16^

**General inputs**: General inputs on the proportion of hospitalized patients, deaths among ambulatory care patients, and the duration of COVID-19 symptoms from onset among vaccinated and unvaccinated patients were sourced from Japanese government data and COVID-19 registry data in Japan **(Table 1)**.^3,20–26^ General inputs, including the proportion of hospitalized patients and deaths, were derived from the averages among the general patient population, which encompasses patients with risk factors for severe COVID-19.

**Table 1.** Model Inputs

**Input Parameter**	**Value**	**Consideration of Omicron Era**	**Consideration of COVID-19 Vaccine**	**Source (Reference No.)**
Epidemiology				
Vaccinated patients with high-risk factors for severe condition	94.17%	Yes		Calculated based on 20
Hospitalization rates (unvaccinated/vaccinated)	25.41%/1.77%			Calculated based on 21
Mortality rates in ambulatory care patients (unvaccinated/vaccinated)	0.03%/0.03%		Same value used for unvaccinated and vaccinated as Japanese data unavailable	Calculated based on 22, 23
Duration of COVID-19 symptoms in outpatients (unvaccinated/vaccinated)	7 days/7 days		Same value used for unvaccinated and vaccinated as Japanese data unavailable	3
ICU admission rate	10.70%	No	No	24
Percentage of MV use on ICU admission	64.54%			
Mortality rates in inpatient (general wards/ICU [without MV/ICU [with MV])	3.19%/11.80%/11.80%	Yes	Yes	Calculated based on 25, 26
Treatment effect - Reduction of hospitalization or death				
Base case (NMV/r vs molnupiravir)				
NMV/r	88.2%	No	No	Estimated by MAIC, relative risk reduction to SoC
Molnupiravir	30.7%	No	No	Estimated by MAIC, relative risk reduction to SoC
Scenario case (NMV/r vs SoC)				
NMV/r	73.8%	No	Yes	29, relative risk reduction to SoC
SoC	0%	No	No	29, relative risk reduction to SoC
Utility				
Baseline utility	0.877	Unknown because it is not mentioned in the data source	The referred study includes both vaccinated and unvaccinated patients but did not stratify 2 patients for COVID-19 infected.	30
Disutility values for ambulatory care patients or patients admitted to general wards	-0.056			
Disutility value for ICU patients without MV or with MV	-0.284			
Disutility value for PACS	-0.027	Unknown because it is not mentioned in the data source	Unknown because it is not mentioned in the data source	31, subtracting the baseline QoL value (0.877) from the QoL value (0.850)
Costs				
Outpatient treatment costs	JPY 26 927 (US $190.22)	Yes	Yes	Hospital information data (EHR) in Japan developed by IQVIA Japan
Hospitalization costs for general wards	JPY 71 515 (US $505.19)/day			
Hospitalization costs with ICU admission (without MV)	JPY 193 598 (US $1367.60)/day			
Hospitalization costs with ICU admission (with MV)	JPY 324 254 (US $2290.57)/day			
Length of stay in general wards	8.76 days			
Length of stay in ICU patients (without MV)	10.90 days			
Length of stay in ICU patients (with MV)	9.78 days			
Medication cost of NMV/r	JPY 99 028/US 699.54(packcost:JPY19 805.5(US139.91) 600 mg [two 300 mg] for nirmatrelvir and 200 mg [two 100 mg] for ritonavir taken for 5 days)	NA	NA	32
Medication cost of molnupiravir	JPY 86 596/US 611.73(packcostfor200mg:JPY2164.9(US15.29) 800 mg [four 200 mg capsules] taken for 5 days)	NA	NA	
Administration cost of NMV/r and molnupiravir	JPY 680 (US $4.80)	NA	NA	

Abbreviations: ICU, intensive care unit; JPY, Japanese yen; MV, mechanical ventilation; NA, not applicable.

**Clinical efficacy**: Two different efficacy estimates were used for NMV/r, depending on the comparator under consideration. First, to inform the effectiveness of NMV/r and molnupiravir, a systematic literature review (SLR) was conducted to obtain the clinical efficacy of NMV/r and molnupiravir in patients with COVID-19 until June 2023. The SLR identified 2 pivotal studies, namely, EPIC-HR and MOVe-OUT, evaluating the efficacy and safety of NMV/r and molnupiravir, respectively **(Supplementary Figure S1).**^4,27^ Second, as direct head-to-head clinical trials comparing the clinical efficacy of NMV/r and molnupiravir were absent, anchored matching-adjusted indirect comparison (MAIC) approach was adopted.^28^ The Japanese HTA guideline recommends the use of randomized controlled trial (RCT) data to inform cost-effectiveness evaluations.^15^ Consequently, 2 pivotal RCTs were utilized for the MAIC. This is a novel technique allowing for a robust indirect comparison, by re-weighting individual patient data from one study to the baseline summary statistics of another, to provide greater adjustment for observed trial differences compared with conventional meta-analytic methods. From the clinical and statistical perspective, the potential effect modifiers to be used for adjustment were defined as time from symptom onset, comorbidities, obesity status, age, baseline serology status, race, sex, and baseline COVID-19 severity (**Supplemental Description 1**). The clinical efficacy end points of NMV/r (EPIC-HR) and molnupiravir (MOVe-OUT) was COVID-19–related hospitalization or death through day 28 and COVID-19– related hospitalization (defined as ≥24 hours of acute care in a hospital or any similar facility) or death through day 29, respectively. They were compared among nonhospitalized adults with mild to moderate COVID-19 at high risk of progression to severe disease based on the MAIC method using individual patient data from EPIC-HR trial modified intention-to-treat (mITT) population without missing values of primary baseline characteristics (n = 1776) and aggregate data from MOVe-OUT mITT population (n = 1408) (**Supplementary Figure S2** and **Supplementary Table S1)**. The effective sample size (ESS) for the EPIC-HR study after matching was 223. **Supplementary Figure S3** shows the distribution of weights of each patient in EPIC-HR accounting for the difference in distribution of baseline characteristics for the studies used for the indirect comparison.

In both trials, there was a high percentage of missing values for COVID-19 variants (EPIC-HR trial, 33.28%; MOVe-OUT trial, 44.32%; **Supplemental Description 1**). Additionally, the distribution of variants differed significantly between the trials. The analysis including COVID-19 variants as matching variables was conducted as a sensitivity analysis in two ways:

- *Sensitivity analysis 1*: Patients with missing variant information were excluded, and the remaining patients with available variant information were adjusted using the variants as matching variables.
- *Sensitivity analysis 2*: Patients with missing variant information in the EPIC-HR trial were assumed to have the Delta variant, and the groups were adjusted using the variants as matching variables.

Additional details are provided in **Supplemental Description 1.**

Using MAIC, NMV/r was shown to reduce the proportion of COVID-19–related hospitalization or all-cause death by 88.16% (= 1 − 0.0118/0.1000) compared with the placebo group. However, molnupiravir was shown to reduce this proportion by 30.68% (1 − 0.0635/0.0916) compared with placebo, as reported by Jayk Bernal et al.^27^ The proportion of COVID-19–related hospitalization or all-cause death in the placebo group in EPIC-HR before matching was 6.66%. The proportion of COVID-19-related hospitalization or all-cause death in the placebo group in EPIC-HR after matching was 10.00% and 9.16% in MOVe-OUT, which was similar, and it indicates that the adjustment between EPIC-HR and MOVe-OUT was effective. This shows that despite differences in variants and vaccination status between the two trials, the results were same. Comparing the reduction in COVID-19–related hospitalization or all-cause death in the NMV/r group and molnupiravir group, it was -6.02% (= -8.83 − [-2.81]) (95% confidence interval [CI]: -8.83 to -3.17), indicating a statistically significant additional benefit for NMV/r over molnupiravir (**Supplementary Table S2**). Patients can die at any point in time due to general mortality reported in Japan and is accounted for in the model.^19^

Both sensitivity analysis 1 and sensitivity analysis 2 demonstrated additional efficacy of NMV/r compared with molnupiravir with a statistically significant difference, but the ESS in the entire mITT1 population of the EPIC-HR trial was significantly reduced to 23 (**Supplemental Description 2**). Regardless of whether variants were used as matching variables, NMV/r was shown to reduce the proportion of COVID-19–related hospitalization or all-cause death compared with molnupiravir.

The second efficacy analysis estimates the clinical efficacy of NMV/r and SoC. The treatment effect of reduction of hospitalization/death for this scenario analysis was sourced from Leister-Tebbe et al,^29^ and patients who were vaccinated or seropositive from EPIC-HR and EPIC-standard risk trials were used. This resulted in a reduction in the number of hospitalizations and/or reduction in the number of deaths for NMV/r by 73.80% (= 1 − {4/807}/{15/793}]) and 0% for SoC.

**Utilities**: The utility values used in this analysis were those estimated by Kamata et al using the JASTIS database and an online survey.^30^ At baseline, the utility value of 0.877 was used for patients without COVID-19, and disutility values were defined as the change from baseline in utility values as described below. The disutility values of outpatients or hospitalized patients in general beds were calculated by subtracting the baseline utility value from the utility value of patients with COVID-19 who did not require oxygen therapy (0.821), which was -0.056 (0.821 – 0.877). Because Kamata et al reported only the utility values of patients hospitalized in general beds, using the same utility value for outpatients and hospitalized patients in general beds was thought to be a more conservative analysis for NMV/r, which may result in a greater reduction in hospitalizations. Therefore, we used the same utility value for outpatients and hospitalized patients in general beds, which was also supported by the clinical expert. The utility value for patients admitted to the ICU (with or without the use of a ventilator) was calculated by subtracting the baseline utility value from the utility value for patients with COVID-19 who required oxygen therapy (0.593), which was -0.284 (0.593–0.877). Because Kamata et al did not report two separate utility values, one for those admitted to the ICU and those on a ventilator, we considered that patients admitted to the ICU were equivalent regardless of whether they were on a ventilator, which was also supported by the clinical expert. The PACS utility value was calculated by subtracting the baseline utility value (0.877) from the utility value (0.850) of patients with persistent COVID-19 symptoms for more than 4 weeks. The change in utility value was -0.027 (0.850–0.877), which was estimated from the questionnaire survey by Tsuzuki et al^31^ (**Table 1).**

**Costs and length of stay**: To estimate treatment costs associated with COVID-19, we used hospital system data (HIS data), which includes electronic medical records (EMR) and prescription data managed by IQVIA Solutions Japan G.K. The HIS data consisted of 23 facilities (21 Diagnosis Procedure Combination hospitals and 2 clinics) with a cumulative net number of 1.93 million patients as of February 2023. A few treatments for COVID-19, such as neutralizing antibody drugs, are not generally available, and prescription data cannot supplement the actual treatment status of patients for such treatments. However, since HIS data also uses EMR data, it is possible to capture the actual treatment status of patients more accurately, including identifying patients prescribed neutralizing antibody drugs. Patients were identified using EMR and medical receipt information in the HIS data, and the costs of treating patients with COVID-19 were estimated from the medical receipt information. Costs related to illness such as outpatient costs, hospitalization costs, and medication costs were included and all the cost inputs were sourced from hospital information data in Japan (**Table 1**).^32^

For the cost analysis, we conducted a retrospective cohort study of patients with risk factors for severe COVID-19 who were treated with NMV/r and molnupiravir after the Omicron strain dominance period. Patients enrolled in the cohort were followed longitudinally during the study period from 2015 until the start of the analysis (February 2023). The patient identification period was from January 2022 onward during the Omicron strain dominance period. We also included a 1-year look-back period from the month of confirmed diagnosis of COVID-19 (index date) during the patient enrollment period to identify risk factors for severe COVID-19.

Risk factors for COVID-19 severity were defined according to the *COVID-19 Medical Care Handbook, Version 9.0.*^33^ Because outpatient and inpatient treatment costs were required as parameters for the model, their analyses were conducted separately (**Supplementary Figures S4 and S5**). Inpatient treatment costs were classified into three subcategories: general hospital beds, ICU (without ventilator), and ICU (with ventilator).

Patients who had a confirmed diagnosis of COVID-19 (ICD-10: U07.1) during the patient identification period (January 2022 or later) and patients with risk factors for severe COVID-19 during the index date or look-back period were included. Prescription of neutralizing antibodies (medications related to sotrovimab, casirivimab/imdevimab, tixagevimab/cilgavimab in EMR) during the study period were excluded. Prices of medical procedures and drugs were adjusted to recent prices (April 2023) (**Table 1**).

**Model verification and validation**: The model with the input parameters was validated by a clinical expert in Japan. The clinical expert is Japanese physician specializing in infectious disease who works at an acute care hospital in Japan. Additional assumptions upon which the model was based are described in **Supplementary Table S3**.

**Sensitivity and scenario analysis**: For one-way sensitivity analysis, the variation range of each parameter in the one-dimensional sensitivity analysis was determined using ±20% of the parameter value. For the probabilistic sensitivity analysis, we performed 500 simulations to assess the uncertainty of the model by randomly sampling the values of each parameter from a predefined probability distribution for each parameter.

In the scenario analysis, NMV/r was compared with SoC instead of molnupiravir. The treatment effect of reduction of hospitalization/death for this scenario analysis was sourced from Leister-Tebbe et al^29^; details can be found above. Because the same utility values for hospitalized patients in general wards were used for outpatients,^30^ a scenario analysis was done using the disutility values as informed by Goswami et al.^34^

Also, the parameter variation in the settings for PACS was set to 5 years from the first year of COVID-19 symptom onset due to the limited extant research on long-term PACS. In another scenario regarding the efficacy of NMV/r and molnupiravir, hospitalization, and death were treated as separate endpoints in the model, but the additional benefit was confirmed by the composite endpoint of hospitalization and death. The proportion of COVID-19–related hospitalizations was not reported in the MOVe-OUT study; hence, so it was not possible to compare it with the endpoints in the EPIC-HR study. Therefore, in the base case analysis, the reduction in the proportion of hospitalizations and the reduction in the proportion of deaths were applied with the same values in the model, but in the scenario analysis, considering that all-cause death was comparable between the EPIC-HR and MOVe-OUT studies, the parameter for the reduction in the proportion of deaths in the model was substituted with the endpoint of all-cause death. The following percentage reductions in death were used: NMV/r group, 100.00%; molnupiravir group, 89.05%. Additionally, in the scenario analysis, NMV/r was compared with placebo.

## RESULTS

### Base Case of NMV/r vs Molnupiravir

The base case results over a lifetime time horizon showed that the total treatment costs for NMV/r and molnupiravir were ¥6 248 014 vs ¥6 245 829 (US $44 136.86 vs $44 121.42), respectively. The incremental QALY per case of NMV/r compared with molnupiravir was 0.013, and the incremental cost was ¥2185 (US $15.44). The incremental cost-effectiveness ratio was ¥164 934 (US $1165.12) per QALY gained, which was lower than the threshold in Japan (¥5 000 000/QALY [US $35 320.71/QALY]) (**Table 2**).

**Table 2.** Base Case Analysis

**Intervention/Comparator**	**LY**	**Incremental LY**	**QALY**	**Incremental QALY**	**Cost (¥[$]/QALY)**	**Incremental Cost (¥[$]/QALY)**	**ICER (¥⁠[$]/QALY)**
Nirmatrelvir-ritonavir	17.963	0.016	15.752	0.013	¥6 248 014 ($44 136.86)	¥2185 ($15.44)	¥164 934 ($1165.12)
Molnupiravir	17.947		15.739		¥6 245 829 ($44 121.42)		

Abbreviations: ICER, incremental cost-effectiveness ratio; LY, life years; QALY, quality-adjusted life years.

### One-Way Sensitivity Analysis

**Figure 2.**
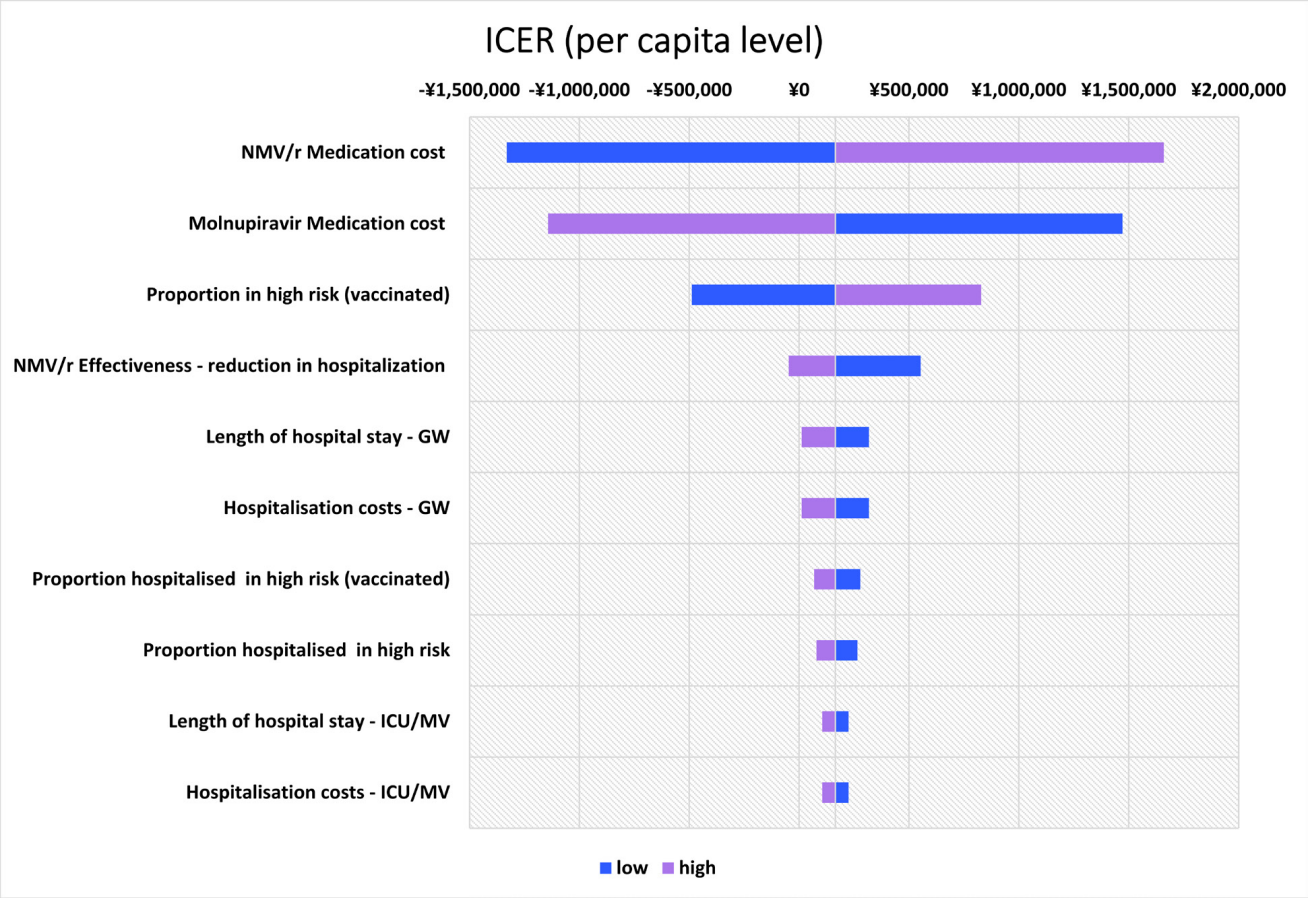
Tornado Diagram of One-Way Sensitivity Analysis Comparing NMV/r with Molnupiravir, Showing Impact on ICER Abbreviations: GW, general ward; ICER, incremental cost-effectiveness ratio; ICU/MV, intensive care unit/mechanical ventilation; NMV/r, nirmatrelvir-ritonavir..

The top 10 parameters with the greatest impact on the ICER using the one-way sensitivity analysis are shown as a tornado diagram in **Figure 2**. The parameters with the greatest impact on the ICER were NMV/r medication cost, molnupiravir medication cost, and the proportion in high risk (vaccinated) individuals. In the one-way sensitivity analysis using the upper limit of NMV/r medication cost, the ICER was ¥1 659 761 /QALY (US $11 724.79 /QALY). All the results showed that the ICER was less than ¥5 000 000/QALY (US $35 320.71/QALY).

### Probabilistic Sensitivity Analysis

**Figure 3.**
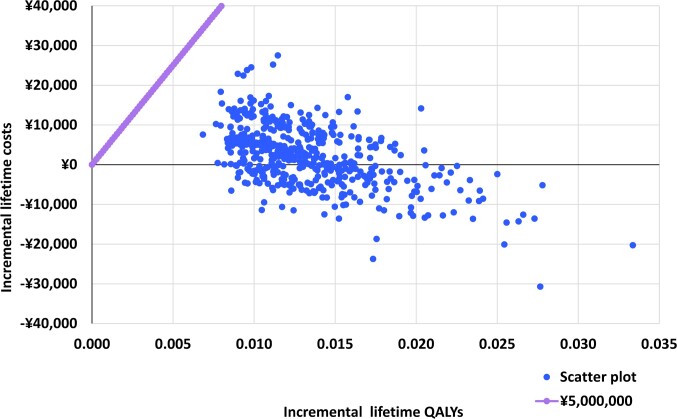
Probabilistic Sensitivity Analysis Scatterplot Comparing NMV/r with Molnupiravir Abbreviations: ICER, incremental cost-effectiveness ratio; NMV/r, nirmatrelvir-ritonavir; QALY, quality-adjusted life year. Note: The blue dots are iterations, and the magenta dot-line is the willingness to pay threshold (¥5 000 000 per QALY gained).

The differences in costs and health outcomes between NMV/r and molnupiravir for each simulation are presented in a probabilistic sensitivity analysis (PSA) scatter plot (**Figure 3**). The results of the probabilistic sensitivity analysis showed that NMV/r was cost-effective in all PSA simulations. Some (37.80%) of the simulation results were in the fourth quadrant of the plot, where NMV/r was dominant compared with molnupiravir. In addition, the result of the cost-effectiveness acceptability curve showed that the probability that the ICER was below the WTP of ¥5 000 000 /QALY (US $35 320.71/QALY) was 100.00% (**Supplementary Figure S6**).

### Scenario Analysis

A scenario analysis comparing NMV/r with SoC showed that the ICER for NMV/r vs SoC was ¥3 646 821/US $25 761.66 per QALY gained (**Table 3**). The deterministic sensitivity analysis was done, and the top 10 most influential parameters ranked from most to least influential are shown in the tornado plot for ICERs (**Supplementary Figure S7**). Nirmatrelvir/ritonavir medication cost, the proportion of hospitalized at high risk (vaccinated), and the proportion of hospitalized at high risk (unvaccinated) were the three parameters with the greatest impact on the ICER. The probability of ICER below the WTP was 78.00% at the WTP threshold in Japan (¥5 000 000/QALY [US $35 320.71/QALY])) as shown in the cost-effectiveness acceptability curve (**Supplementary Figure S8** and **Supplementary Figure S9**).

**Table 3.** Scenario Analysis Results Comparing NMV/r with SoC

**Intervention/⁠Comparator**	**LY**	**Incremental LY**	**QALY**	**Incremental QALY**	**Cost (¥[$]/QALY)**	**Incremental Cost (¥[$]/QALY)**	**ICER (¥[$]/QALY)**
Nirmatrelvir-ritonavir	17.961	0.029	15.751	0.025	¥6 251 137 ($44 158.92)	¥89 570 ($632.73)	¥3 646 821 ($25 761.66)
SoC	17.932		15.726		¥6 161 567 ($43 526.19)		

Abbreviations: ICER, incremental cost-effectiveness ratio; LY, life years; QALY, quality-adjusted life years; SoC, standard of care.

In another scenario analysis, when the impact of PACS was changed from 1 year to 5 years, the ICER was ¥165 899 (US $1171.93) per QALY gained. The scenario case results, using Goswami disutility over a lifetime time horizon, showed that NMV/r had an increased incremental QALY per case of 0.013 compared with molnupiravir. The ICER was ¥3 614 387 (US $25 532.54) per QALY gained. Comparing SoC when using Goswami disutility, the incremental QALY per case was increased (0.025) and the ICER was ¥3 614 387 (US $25 532.54) per QALY gained.

Also in the scenario analysis, when the parameter for the reduction in the proportion of deaths in the model was substituted with the endpoint of all-cause death, the ICER was dominant.

## DISCUSSION

The objective of this study is to estimate the cost-effectiveness of NMV/r compared with molnupiravir in patients with mild to moderate COVID-19 who have risk factors for severe COVID-19 from a Japanese payer perspective. This is the first study to compare NMV/r with molnupiravir directly using the Carlson et al model adaptation^13^ and individual patient data from one study using the MAIC approach. Additionally, we also assessed the cost-effectiveness of NMV/r compared with SoC in the scenario analysis. The study showed that higher QALYs (15.752 vs 15.739) and higher total costs (¥6 248 014 vs ¥6 245 829 [US $44 136.86 vs $44 121.42]) when comparing NMV/r with molnupiravir. The incremental cost-effectiveness ratio was ¥164 934/US $1165.12 per QALY gained, which was lower than the threshold in Japan. The scenario analysis results showed higher costs accruing to patients on NMV/r compared with SoC (¥6 251 137/US $44 158.92 vs ¥6 161 567/US $43 526.19). However, NMV/r gained 0.025 additional QALYs, generating an ICER of ¥3 646 821/US $25 761.66 per QALY gained. This study used different data sources for estimating treatment effects in reducing hospitalizations and deaths for molnupiravir and SoC. Hence, the costs and QALYs were different between base case and scenario analysis.

Molnupiravir in the MOVe-OUT study suggested a 30% reduction in hospital admissions and deaths compared with placebo among unvaccinated and nonhospitalized patients with COVID-19. Although the endpoints and study design of EPIC-HR and MOVe-OUT trials were different, the effectiveness of NMV/r was consistent. A network meta-analysis by Lai et al using the results of the EPIC-HR and MOVe-OUT trials with similar endpoints to the MAIC of this analysis showed a significantly lower proportion of COVID-19–related hospitalization or all-cause death for NMV/r compared with molnupiravir (odds ratio=0.17 [95% CI: 0.07-0.39]).^35^ In the PLATCOV study, which was an open-label, phase 2, randomized, controlled trial, NMV/r showed an 84% (95% CI: 54 to 119) faster viral clearance rate and molnupiravir—a 37% (95% CI: 16-65) faster viral clearance relative to the control (no study drug) group.^36^

In this study, HIS data comprising EMR and prescription data were used for the cost analysis, and appropriate patient selection was performed for COVID-19 treatment, excluding treatments that are not generally available, such as neutralizing antibody drugs. The validity of our results was also supported by the clinical expert. In a cost analysis study by Uno et al, the cost of COVID-19 hospitalization in a single hospital was analyzed; it excluded COVID-19 drug costs, so it was not possible to make a general comparison with our model.^37^ However, the study reported the median length of hospital stay according to the severity of COVID-19 (all patients: 10 days; mild: 8 days; moderate I: 9 days; moderate II: 12 days; severe: 17 days). In our analysis, the average length of hospital stay was used in the model, but the median length of hospital stay was also calculated, similar to the above study, with 10 days for general beds, 12 days for ICUs without ventilators, and 14 days for ICUs with ventilators.

Our results were consistent with previous cost-effectiveness studies that showed NMV/r was cost-effective. According to the special assessment of outpatient treatments for COVID-19 by the ICER, NMV/r showed an ICER of US $21 000 per QALY gained and molnupiravir an ICER of US $61 000 per QALY gained compared with SoC.^12^ A cost-effectiveness study conducted by Wai et al reported ICERs of outpatient and inpatient settings for NMV/r vs SoC and molnupiravir vs SoC. The cost-effectiveness of NMV/r was larger than that of molnupiravir, as the ICERs for NMV/r were lower compared with molnupiravir, in both the outpatient (US $331 105.27 vs US $493 345.09) and inpatient (US $-5502.53 vs US $2629.08) settings.^38^ Another CEA study comparing NMV/r and best supportive care, conducted in Japan, showed NMV/r was more cost-effective than the best supportive care, which was consistent with our study. Additionally, the study compared cost-effectiveness among different age groups, and ICERs were lower among older age groups (≥60 years) compared with younger age groups, suggesting NMV/r was cost-effective in this age group.^9^ However, our study did not evaluate ICERs for patients of different age groups.

This study has several limitations. First, in MAIC analysis, all potential effect modifiers assumed and available for analysis were used. However, there is still a possibility that potential effect modifiers were not available and that applying MAIC did not fully control for possible bias in estimating the proportion of COVID-19 hospitalizations or all-cause deaths. As such, when the EPIC-HR trial was conducted from July to December 2021, the Delta variant was the predominant COVID-19 variant. When the MOVe-OUT trial was conducted from May to November 2021, the Alpha and Gamma variants were predominant; however, they overlapped with the Delta variant’s predominant time frame. Moreover, the rates of hospitalization and death during the periods when Alpha and Delta strains were predominant remained largely unchanged.^39^ Additionally, sensitivity analyses of MAIC using variants as matching variables showed NMV/r reduced the proportion of COVID-19–related hospitalizations or all-cause deaths compared with molnupiravir.

Second, in cases where the variable definitions did not directly correspond between the EPIC-HR and MOVe-OUT trials for the matching variables, the definitions were adjusted or substituted to create variables that were similar. However, these efforts may not fully align these definitions, which is possible that the variables were not matched correctly.

Third, adjustment by MAIC reduced the ESS of the EPIC-HR study mITT1 population by 87.44%, from 1776 to 223 cases. This suggests that there is little overlap in the distribution of patient characteristics between the EPIC-HR and MOVe-OUT trials and that the adjusted data rely on relatively few individual data. On the other hand, a previous study on ESS in MAIC reported that ESS was reduced by an average of 80% (range: −98% to 57%) from the original sample size in the MAIC cases considered. Thus, the results of this analysis did not show a significant decrease in ESS compared with previous studies.^40–42^ In addition, despite the decrease, the original clinical trial had a large number of cases, and the present analysis secured several hundred ESS cases.^43^

Fourth, this analysis utilized utilities derived from studies conducted on the Japanese population,^30,31^ in accordance with the Japanese HTA guideline.^15^ However, the utilities did not fully align with the health states of the model. Consequently, utilities from Goswami et al, which were more congruent with the health states, were employed in the scenario analysis.^34^ The results of the scenario analysis, akin to the base case analysis, affirmed the robustness of the results.

Fifth, the target population in this analysis was conditioned on the prevalence of SARS-CoV-2 variants in Japan (since the Omicron variant epidemic) and vaccination status. However, in the SLR, the only RCTs of NMV/r and molnupiravir that met the inclusion and exclusion criteria were the EPIC-HR and MOVe-OUT studies, which were conducted in unvaccinated subjects before the period of Omicron dominance. During the SLR process, the PANORAMIC study,^44^ which examined the efficacy of molnupiravir, and the study of Liu et al,^45^ which examined the efficacy of NMV/r, were also identified, both of which were conducted during the Omicron strain dominance period and included vaccinated individuals, but the outcomes of neither study met the inclusion criteria for the SLR. The outcomes of the PANORAMIC study were all-cause hospitalization or all-cause death, and the EPIC-HR study did not specify all-cause hospitalization as an outcome, making it difficult to make an indirect comparison using the two studies. On the other hand, for the indirect comparison between the PANORAMIC study and Liu et al study, the distribution of comorbidities in patients differed greatly between the two studies, making it difficult to adjust for patient backgrounds based on aggregate data. In addition, the PANORAMIC study and Liu et al study were conducted in the United Kingdom and China, respectively, so they may not reflect the actual clinical situation in Japan.

In summary, when conducting an indirect comparison of therapeutic drugs at a single point in time for a disease such as COVID-19 infection, where the circumstances surrounding the disease are changing and evidence is being updated rapidly, it is first necessary to select an appropriate trial (RCT) at the time of conducting the SLR, fully considering the consistency of the outcomes and inclusion and exclusion criteria specified in each trial. Furthermore, when conducting an indirect comparison using an RCT selected by the SLR, it is necessary to fully consider whether the effect modifiers in each trial can be used. In addition, it is necessary to reflect changes in the situation regarding the disease in a timely manner, such as the prevalence of mutant strains (Omicron strain and later) and vaccinations, in the analysis results by using various epidemiological data inserted into the CEA model.

In this study, when the parameters that took into account the prevalence of SARS-CoV-2 variants in Japan (since the Omicron strain epidemic) and vaccination status were available, they were used preferentially, although as a result of the SLR, the only RCTs of NMV/r and molnupiravir that met the inclusion and exclusion criteria were the EPIC-HR and MOVe-OUT studies, which were conducted in unvaccinated subjects before the Omicron strain dominance period.

## CONCLUSION

Our cost-utility analysis demonstrates that NMV/r improves quality of life and increases healthcare costs compared with molnupiravir or no treatment. These benefits are gained from reductions in hospitalization and death driven by the higher effectiveness relative to the comparator. As the Japanese government stopped public funding for anti-COVID drugs since April 2024, the findings of this study provide important evidence for the cost-effectiveness of NMV/r from a Japanese payer perspective.

### Disclosures

AY and KM are full-time employees of Pfizer Japan Inc. HM and YK are full-time employees of IQVIA Solutions Japan G.K., which received funding from Pfizer Japan Inc. to undertake the research outlined in this study. YF and MY are full-time employees of Pfizer R&D Japan. TM is a full-time employee of Pfizer Inc. YG reports lecture fees not related to the submitted work from Pfizer Japan Inc., bioMérieux Japan Ltd, Nippon Becton Dickinson Company Ltd., and Gilead Sciences Inc., advisory fees from Pfizer Japan Inc., advisory fees not related to the submitted work from bioMérieux Japan Ltd., and research grants not related to the submitted work from MSD K.K. The authors have no other relevant affiliations or financial involvement with any organization or entity with a financial interest in or financial conflict with the subject matter or materials discussed in the manuscript apart from those disclosed.

### Data availability statement

The data sets generated during and/or analyzed during the current study are available from the corresponding author on reasonable request.

## Supplementary Material

Online Supplementary Material
