# Supplementary material for: Cost-effectiveness Analysis of Nirmatrelvir/Ritonavir Compared with Molnupiravir in Patients at High Risk for Progression to Severe COVID-19 in Japan
Source: J Health Econ Outcomes Res. 2025 Feb 24;12(1):75–85. doi: 10.36469/001c.129067 (PMC11864595; doi:10.36469/001c.129067)
Supplement: Online Supplementary Material [file jheor_2025_12_1_129067_267324.pdf]

Journal of Health Economics and Outcomes Research

Online Supplementary Material

Cost-effectiveness Analysis of Nirmatrelvir/Ritonavir vs Molnupiravir in Patients at High Risk for Progression to Severe COVID-19 in Japan. *JHEOR*. 2025;12(1):75-85. doi:10.36469/jheor.2025.129067

Table S1. Baseline Characteristics **2**

Table S2. COVID-19–Related Hospitalization or All-Cause Death Before/After Matching **3**

Table S3. Assumptions of Cost-Effectiveness Analysis **3**

Figure S1. PRISMA Diagram **4**

Figure S2. Conceptual Figure of Anchored MAIC Comparing NMV/r to Molnupiravir **4**

Figure S3. Distribution of Weights for EPIC-HR Data in MAIC Analysis **5**

Figure S4. Analysis Design for Outpatient Cost **5**

Figure S5. Analysis Design for Inpatient Costs **6**

Figure S6. Probabilistic Sensitivity Analysis: Cost-Effectiveness Acceptability Curve Comparing NMV/r to Molnupiravir **6**

Figure S7. Tornado Diagram of One-Way Sensitivity Analysis of Scenario Analysis Comparing NMV/r to SoC, Showing the Top 10 Parameters with the Greatest Impact on the ICER **7**

Figure S8. Probabilistic Sensitivity Analysis Scatterplot of Scenario Analysis Comparing NMV/r to SoC **7**

Figure S9. Probabilistic Sensitivity Analysis: Cost-Effectiveness Acceptability Curve Comparing NMV/r to SoC **8**

Supplemental Description 1 **8**

Table S4. Matching Variables for MAIC **8**

Table S5. COVID-19 Disease Severity Definitions of MOVe-OUT[4] **9**

Supplemental Description 2 **10**

Table S6. Proportions of COVID-19 Variants on EPIC-HR and MOVe-OUT Trials **10**

Table S7. Baseline Characteristics in Sensitivity Analysis 1 **11**

Figure S10. Distribution of Weights for EPIC-HR Data in Sensitivity Analysis 1 **12**

Table S8. COVID-19-Related Hospitalization or All-Cause Death Before/After Matching in Sensitivity Analysis 1 **13**

Table S9. Baseline Characteristics in Sensitivity Analysis 2 **13**

Figure S11. Distribution of Weights for EPIC-HR Data in Sensitivity Analysis 2 **15**

Table S10. COVID-19–Related Hospitalization or All-Cause Death Before/After Matching in Sensitivity Analysis 2 **15**

References **16**

This supplementary material has been provided by the authors to give readers additional information about their work.

This is an open-access article distributed under the terms of the Creative Commons Attribution 4.0 International License (CCBY-4.0). View this license's legal deed at http://creativecommons.org/licenses/by/4.0 and legal code at http://creativecommons.org/licenses/by/4.0/legalcode for more information.

Table S1. Baseline Characteristics

Baseline Variable	MOVe-OUT						EPIC-HR							*P* Value vs Original Sample Total[a]	*P* Value vs Matched Sample Total[a]
	AgD Mol (N = 709)		AgD Placebo (N = 699)		AgD Total (N = 1408)		Original Sample N/R (N = 875)		Original Sample Placebo (N = 901)		Original Sample Total (N = 1776)		Matched Sample Total (ESS = 223)		
	n	%	N	%	n	%	n	%	n	%	n	%	%		
Sex															
Male	330	46.5	355	50.8	685	48.7	443	50.6	462	51.3	905	51.0	48.7	.209	1.000
Female	379	53.5	344	49.2	723	51.3	432	49.4	439	48.7	871	49.0	51.3		
Age group															
≤60 years	591	83.4	572	81.8	1163	82.6	725	82.9	717	79.6	1442	81.2	82.6	.330	1.000
>60 years	118	16.6	127	18.2	245	17.4	150	17.1	184	20.4	334	18.8	17.4		
Race															
White only	397	56.0	405	58.0	802	57.0	599	68.5	640	71.0	1239	69.8	57.0	<.001	1.000
Black only	39	5.5	34	4.9	73	5.2	41	4.7	29	3.2	70	3.9	5.2		
Asian only	25	3.5	22	3.2	47	3.3	134	15.3	132	14.7	266	15.0	3.3		
Native American	178	25.1	172	24.6	350	24.9	94	10.7	92	10.2	186	10.5	24.9		
Other	70	9.9	65	9.3	135	9.6	7	0.8	8	0.9	15	0.8	9.6		
Obesity status (BMI ≥30 kg/m^2)															
Yes	535	75.5	507	72.5	1042	74.0	304	34.7	314	34.9	618	34.8	74.0	<.001	1.000
No	174	24.5	192	27.5	366	26.0	571	65.3	587	65.1	1158	65.2	26.0		
Comorbidities															
Diabetes mellitus	107	15.1	117	16.7	224	15.9	94	10.7	97	10.8	191	10.8	15.9	<.001	1.000
Cardiovascular disorders[b]	86	12.1	78	11.2	164	11.6	30	3.4	38	4.2	68	3.8	11.6	<.001	1.000
Time from symptom onset															
≤3 days	339	47.8	335	47.9	674	47.9	601	68.7	589	65.4	1190	67.0	47.9	<.001	1.000
>3 days	370	52.2	364	52.1	734	52.1	274	31.3	312	34.6	586	33.0	52.1		
Baseline serology status															
Positive	136	20.1	146	21.9	282	21.0	446	51.0	450	49.9	896	50.5	21.0	<.001	1.000
Negative	541	79.9	520	78.1	1061	79.0	429	49.0	451	50.1	880	49.5	79.0		
Baseline COVID-19 severity															
Mild	395	55.9	376	53.9	771	55.0	326	37.3	306	34.0	632	35.6	55.0	<.001	1.000
Moderate	311	44.1	321	46.1	632	45.0	549	62.7	595	66.0	1144	64.4	45.0		

Abbreviations: AgD, aggregated data; COVID-19, coronavirus disease 2019; ESS, effective sample size; MOL, molnupiravir; N/R, nirmatrelvir-ritonavir.
[a] *P* values were estimated based on χ^2 test for categorical variables.
[b] Refers to serious heart condition (heart failure, coronary artery disease, or cardiomyopathies) in MOVe-OUT AgD.

Table S2. COVID-19–Related Hospitalization or All-Cause Death Before/After Matching

	Intervention			Placebo			Total ESS (N)	N/R: Placebo (A)	Mol: Placebo (B)	A – B	Lower 95% CI	Upper 95% CI
	Total	Outcome		Total	Outcome							
	N	n	%	N	n	%						
MOVe-OUT AgD	709	45	6.35	699	64	9.16						
EPIC-HR before matching	875	8	0.91	901	60	6.66						
EPIC-HR after matching			1.18			10.00	223	-8.83%	-2.81%	-6.02%	-8.83%	-3.17%

Abbreviations: AgD, aggregated data, CI, confidence interval, COVID-19, coronavirus disease 2019, ESS, effective sample size, MOL, molnupiravir, N/R, nirmatrelvir-ritonavir.

Table S3. Assumptions of Cost-Effectiveness Analysis

Model Variables	Assumptions
Model analysis period	The analysis period was a lifetime to allow for a period of analysis long enough to assess the impact on the costs and effectiveness of the technologies under evaluation.
No. of people treated	Each of the nirmatrelvir-ritonavir and molnupiravir groups included 1000 COVID-19 patients.
Patient characteristics in the model	The subjects of this analysis were COVID-19 patients with risk factors for severe disease who were treated with nirmatrelvir-ritonavir or molnupiravir in clinical practice. When available, data from the period after the predominance of the Omicron strain in Japan and taking into account the presence or absence of vaccination were used preferentially as input parameters. When data on patients with risk factors for severe disease were not available, data consisting of patients aged 60 (or 65) or older were used as the parameter for "patients with risk factors for severe disease."
Long-term post-acute COVID-19 syndrome (PACS) setup	There were limited data and evidence on long-term PACS. Therefore, in the baseline analysis, we considered the impact of PACS only in the first year. In the scenario analysis, we changed the parameters to other than one year and estimated the results.
Efficacy of nilmatrevir-ritonavir and molnupiravir	Although hospitalization and death were treated as separate endpoints in the model, indirect comparisons were made between hospitalization and death, which was the composite endpoint for evaluating additional usefulness. The proportion of hospitalizations related to COVID-19 was not reported in the MOVe-OUT study, so it was not possible to compare it with the endpoints in the EPIC-HR study. Therefore, the reduction in hospitalizations and deaths was applied to the same values as the reduction in hospitalizations and deaths in the model. For this assumption, parameters were changed in a scenario analysis to evaluate the impact.
Adverse event	In both the EPIC-HR and MOVe-OUT studies, the incidence of serious adverse events due to nirmatrelvir-ritonavir and molnupiravir was extremely low (nirmatrelvir-ritonavir: <.1%, molnupiravir: 0%), so the occurrence of serious adverse events was not considered in this analysis. In addition, non-serious adverse events were not considered in this analysis because their impact on efficacy (QOL), costs, and associated outcomes was assumed to be minor.
QOL scores for outpatients or patients admitted to general hospitals	Kamata et al, referring to QOL values.[1] In the previous study, only QOL values for patients admitted to general hospital beds were reported. We thought that using the same QOL values for outpatients and patients admitted to general hospital beds would be a more conservative analysis for nirmatrelvir-ritonavir, which may further reduce hospitalizations. Therefore, we used the same QOL values for outpatients and patients admitted to general hospital beds. This method was also supported by clinical experts.
Patients admitted to the ICU (non-ventilator and ventilator-assisted)	Kamata et al[1], referring to QOL values, did not report QOL values according to ICU and ventilator status, and considered ICU admissions to be equivalent for patients on and off ventilators. This approach was also supported by clinical experts.

Abbreviations: COVID-19, coronavirus disease 2019; ICU, intensive care unit; PACS, post-acute COVID-19 syndrome COVID-19; QOL, quality of life.

Figure S1. PRISMA Diagram

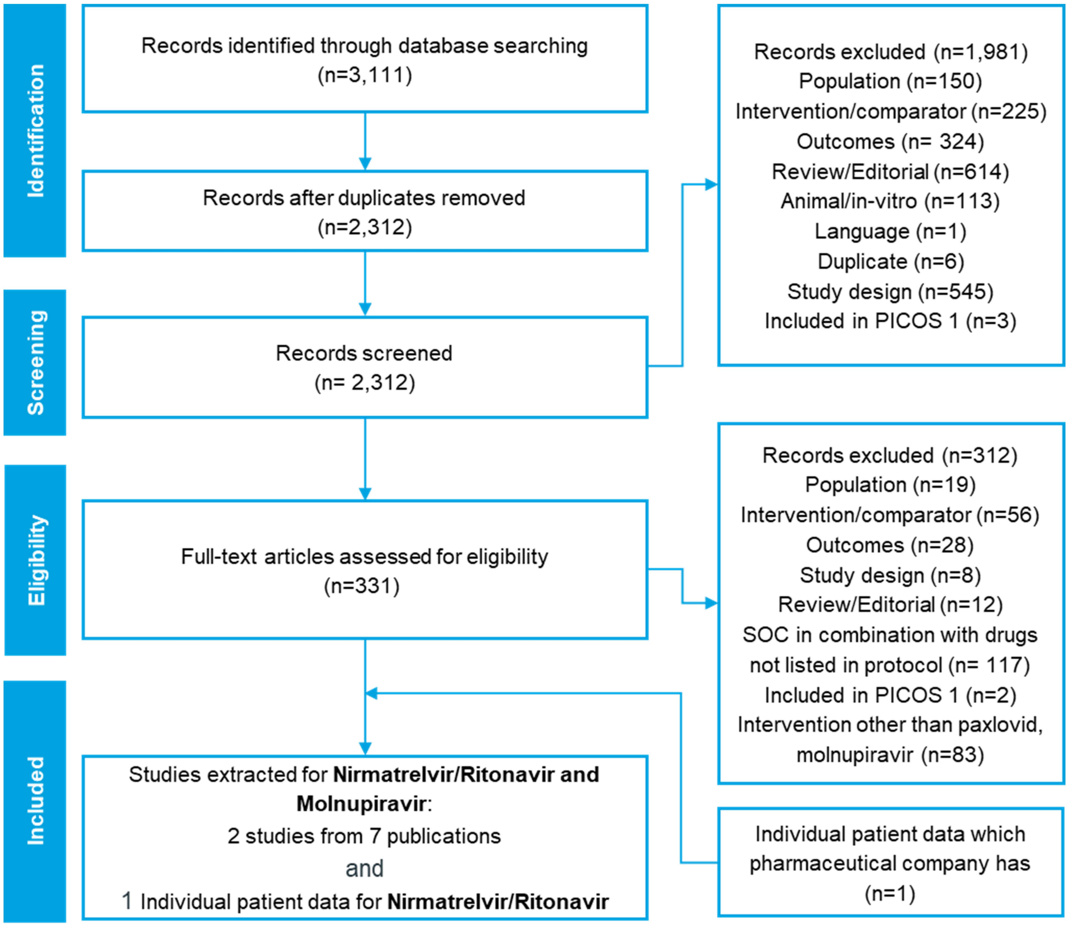

Figure S2. Conceptual Figure of Anchored MAIC Comparing NMV/r to Molnupiravir

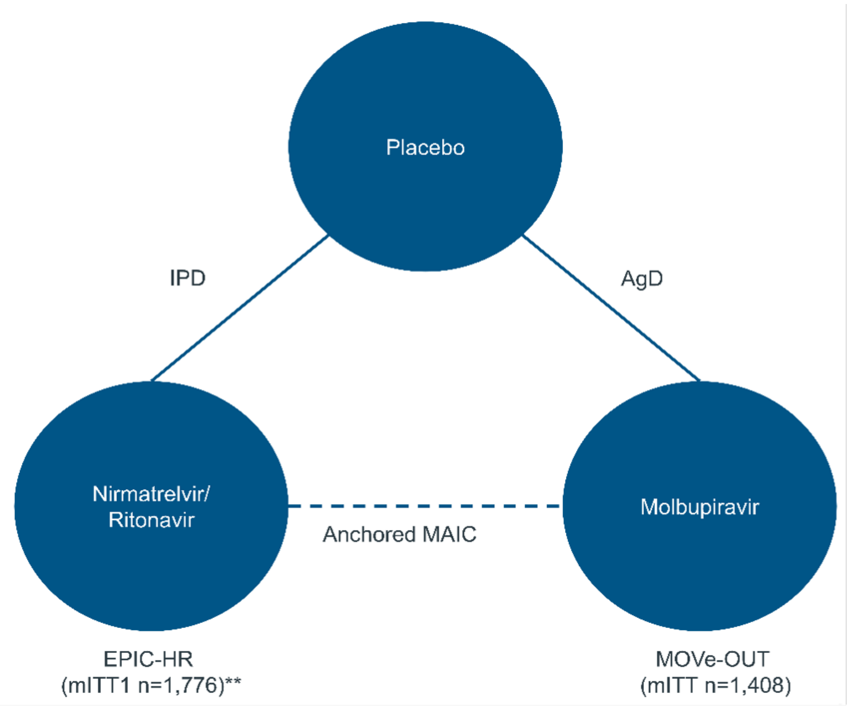

Abbreviations: AgD, aggregated data; IPD, individual patient data; MAIC, matching-adjusted indirect comparison; mITT, modified intention-to-treat; NMV/r, nirmatrelvir/ritonavir.

Figure S3. Distribution of Weights for EPIC-HR Data in MAIC Analysis

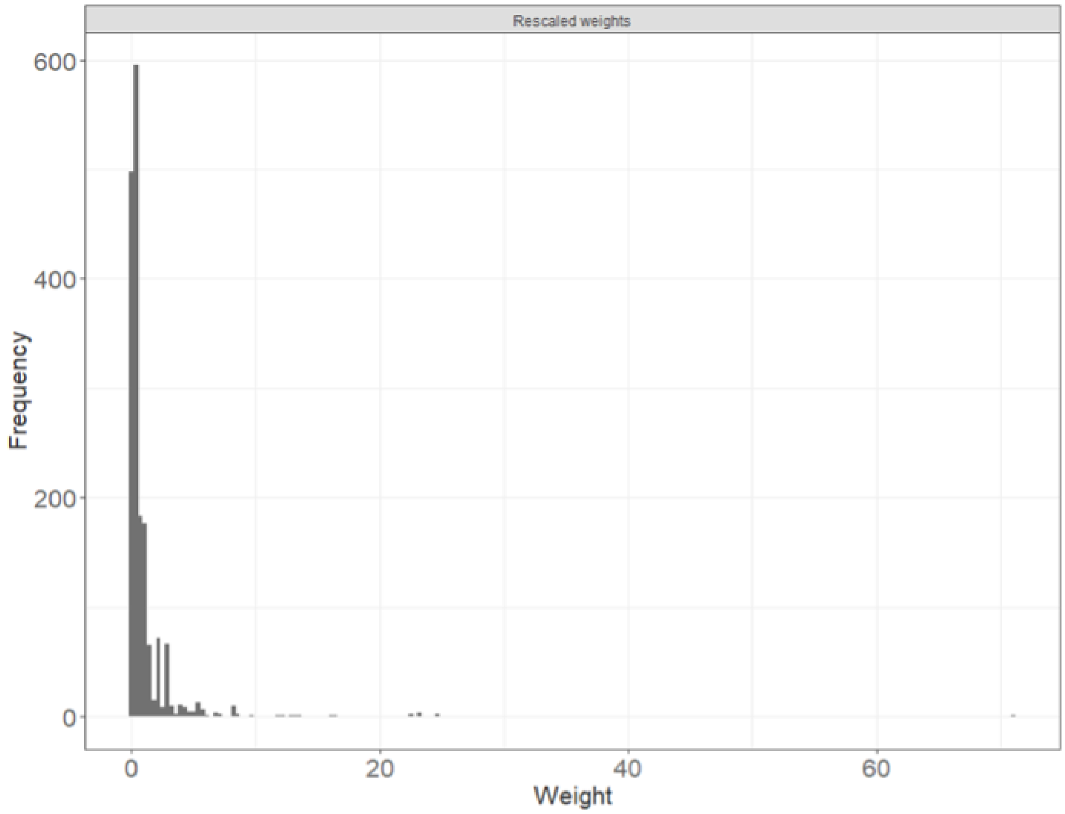

Abbreviation: MAIC, matching-adjusted indirect comparison.

Figure S4. Analysis Design for Outpatient Cost

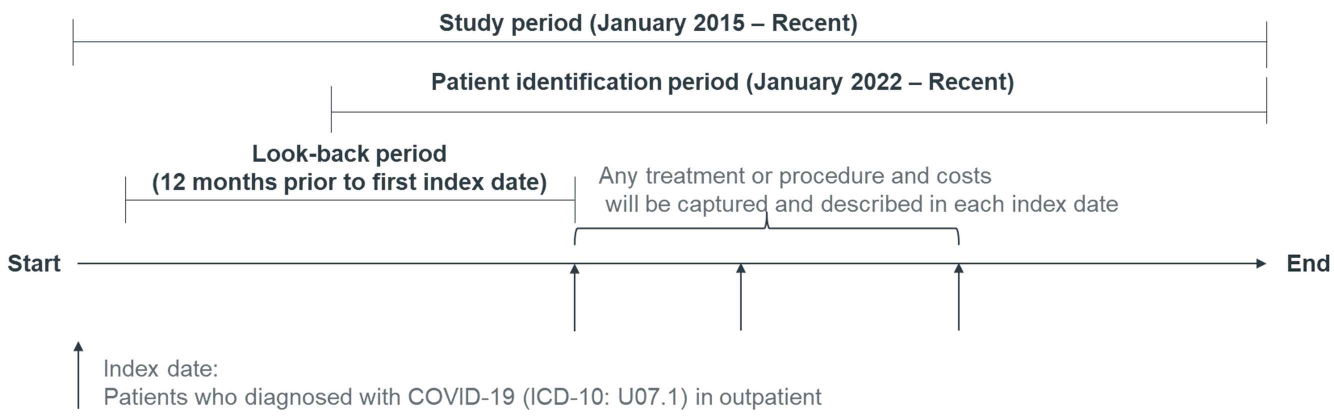

Abbreviations: COVID-19, coronavirus disease 2019; ICD, *International Statistical Classification of Diseases and Related Health Problems*.

Figure S5. Analysis Design for Inpatient Costs

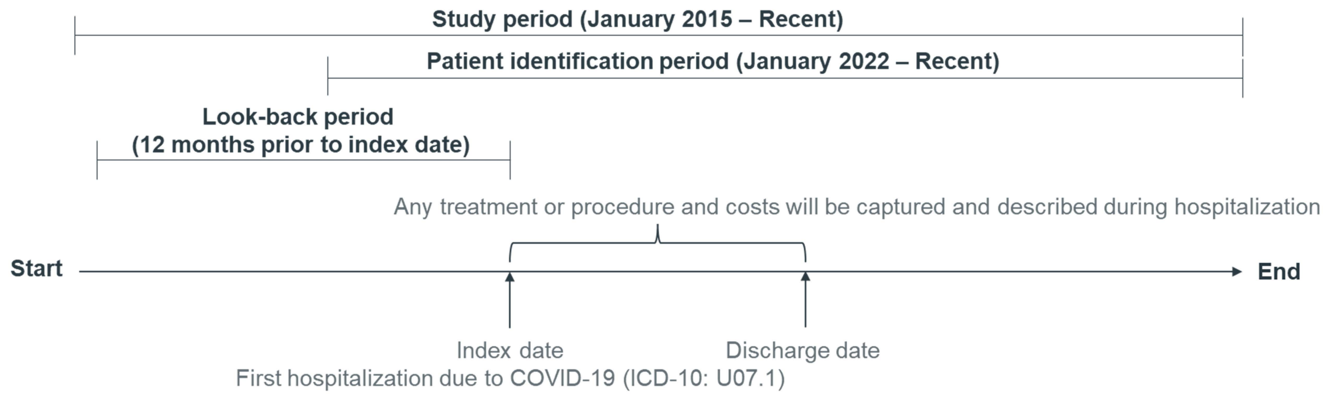

Abbreviations: COVID-19, coronavirus disease 2019, ICD, *International Statistical Classification of Diseases*.

Figure S6. Probabilistic Sensitivity Analysis: Cost-Effectiveness Acceptability Curve Comparing NMV/r to Molnupiravir

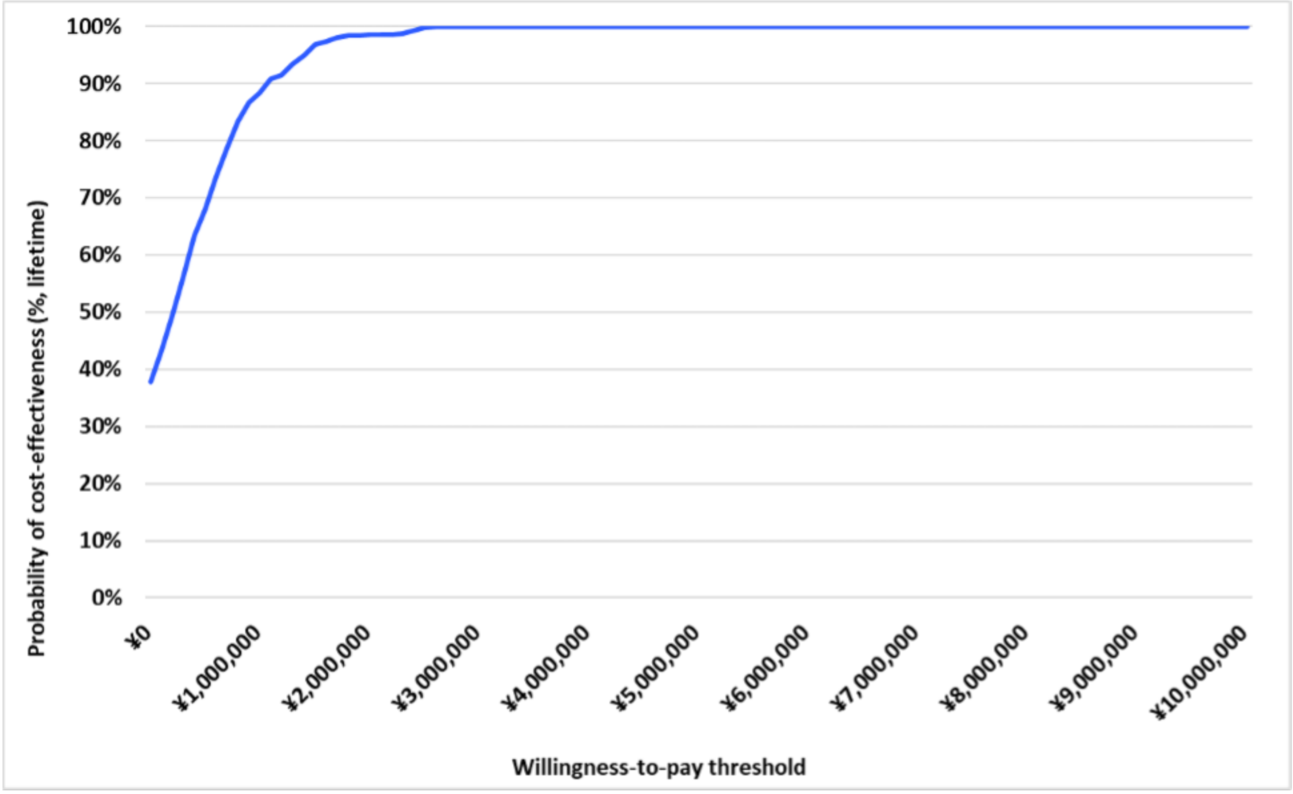

Abbreviation: NMV/r, nirmatrelvir-ritonavir.

Figure S7. Tornado Diagram of One-Way Sensitivity Analysis of Scenario Analysis Comparing NMV/r to SoC, Showing the Top 10 Parameters with the Greatest Impact on the ICER

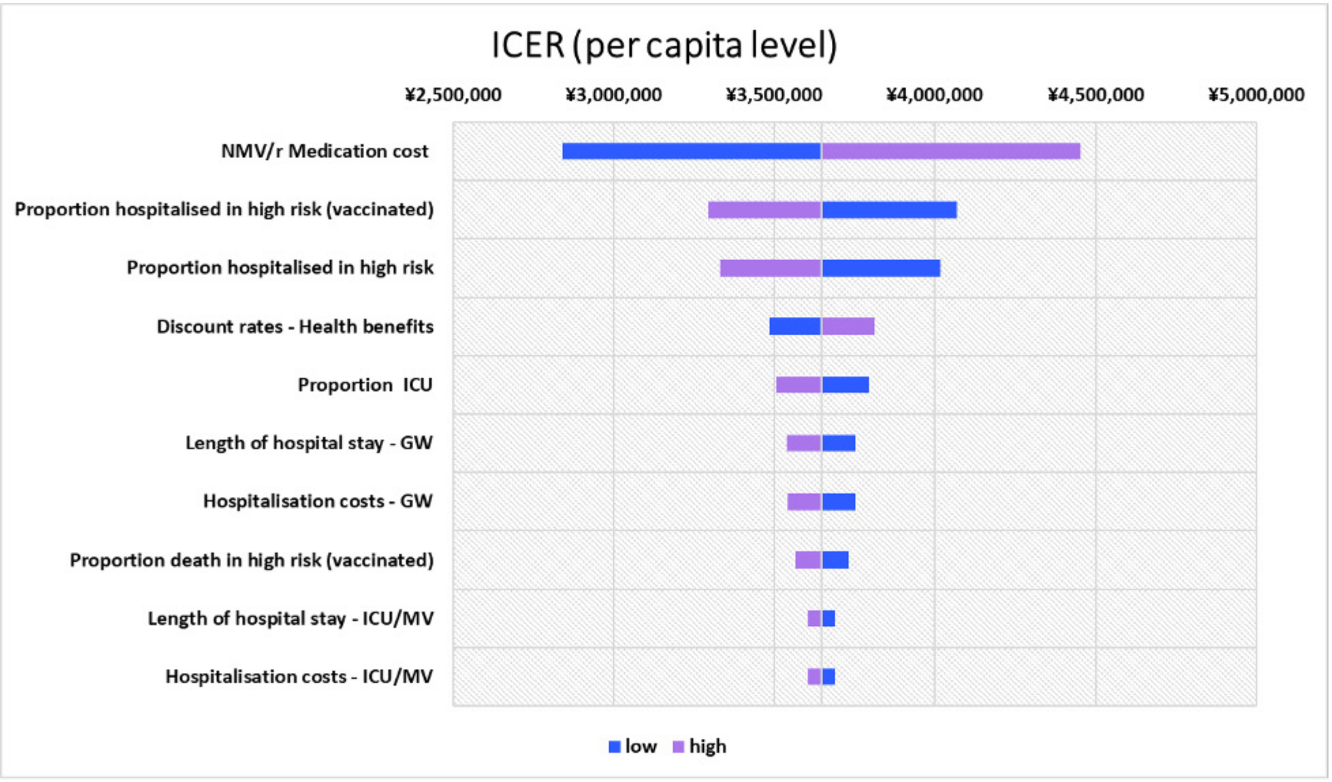

Abbreviations: GW, general ward; ICER, incremental cost-effectiveness ratio; ICU, intensive care unit; ICU/MV, intensive care unit/mechanical ventilation; NMV/r, nirmatrelvir-ritonavir; SoC, standard of care.

Figure S8. Probabilistic Sensitivity Analysis Scatterplot of Scenario Analysis Comparing NMV/r to SoC

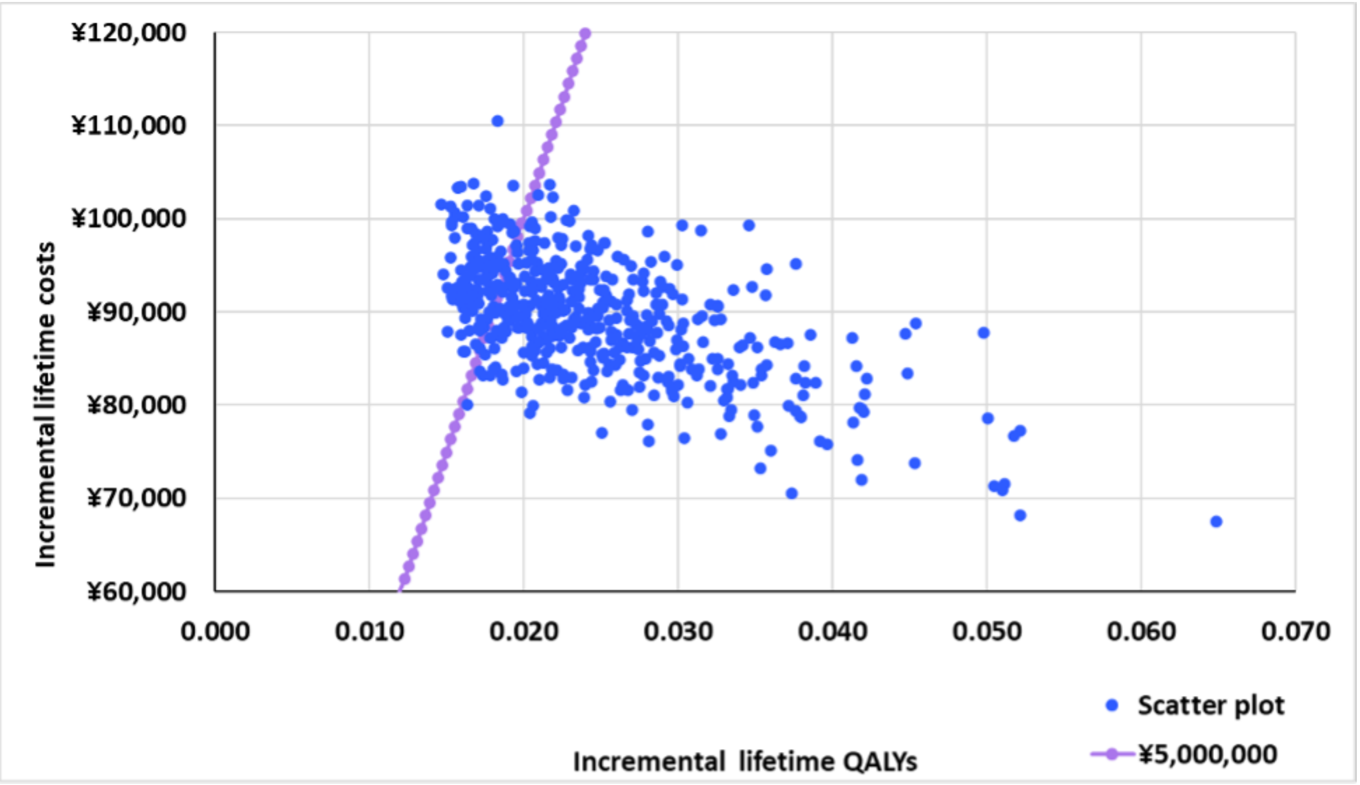

Abbreviations: NMV/r, nirmatrelvir-ritonavir; QALY, quality-adjusted life year; SoC, standard of care.

Figure S9. Probabilistic Sensitivity Analysis: Cost-Effectiveness Acceptability Curve Comparing NMV/r to SoC

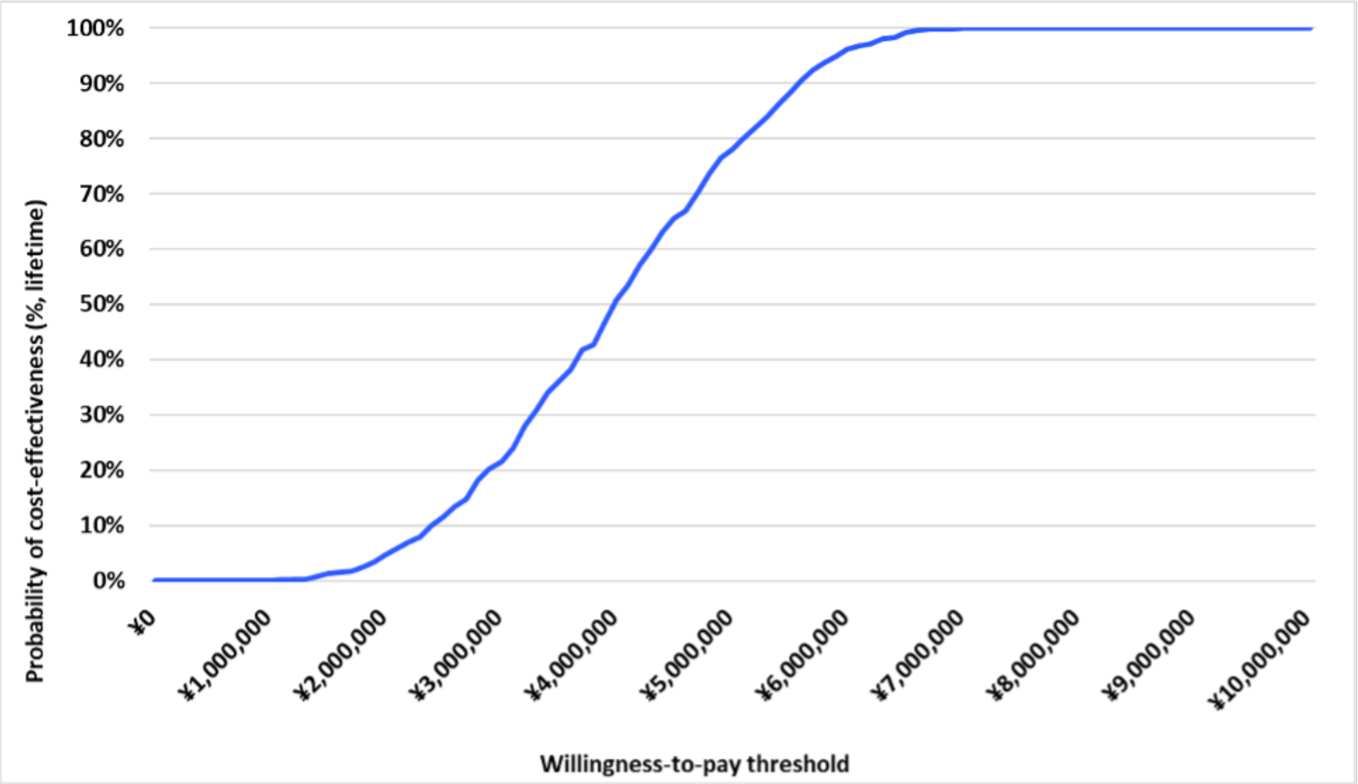

Abbreviations: NMV/r, nirmatrelvir-ritonavir; SoC, standard of care.

Supplemental Description 1

Potential effect modifiers for the primary endpoints (hereinafter referred to as potential effect modifiers) were selected based on existing clinical knowledge and the results of subgroup analyses from the EPIC-HR and MOVe-OUT trials. Although the severity of COVID-19, one of the effect modifiers, was not reported in the subgroup analysis of the EPIC-HR trial, it was included as a potential effect modifier due to the external assessment group at NICE's Multi-Technology Appraisal mentioning that the severity of COVID-19 should be treated as an effect modifier in evaluations involving nirmatrelvir and ritonavir.[2]

Finally, the potential effect modifiers that could be matched between the EPIC-HR trial (Individual Patient Data [IPD]) and the MOVe-OUT trial (aggregate data [AgD]) were examined, and the variables to be used for matching (hereinafter referred to as matching variables) were determined. **Table S4** lists the matching variables selected for this analysis.

Table S4. Matching Variables for MAIC

Variable	Type
Gender (male, female)	Categorical variable
Age (≤60 years, ≥61 years)	Categorical variable
Race (White, Black, Asian, Native American, Other)	Categorical variable
Obesity (BMI <30 kg/m², BMI ≥30 kg/m²)	Categorical variable
Diabetes (with diabetes, without diabetes)	Categorical variable
Coronary artery disease (with coronary artery disease, without coronary artery disease)	Categorical variable
Time of COVID-19 symptom onset (within 3 days, 4 days or more)	Categorical variable
Baseline COVID-19 plasma (with antibodies, without antibodies)	Categorical variable
Baseline severity (mild, moderate)	Categorical variable
<Sensitivity Analysis> COVID-19 variants (Gamma, Delta, Mu, Other)	Categorical variable

Abbreviations: BMI, body mass index; COVID-19, coronavirus disease 2019.

When aligning the matching variables between the EPIC-HR trial and the MOVe-OUT trial, we adjusted for differences in racial classification and the definitions of COVID-19 severity.

1. Reclassification of Racial Categories

Racial classification differed between the two trials as follows:

- **EPIC-HR trial:** White only, Black only, Asian only, Native American, and Other
- **MOVe-OUT trial**: Asian only, Black only, Black-White mixed, Native American only, Native American–White mixed, Native American–Black-White mixed, White only, and Other multiple races

For the purpose of this analysis, the racial categories in the MOVe-OUT trial were reclassified to match those in the EPIC-HR trial as follows:

White only, Black only, Asian only, Native American (including Native American only, Native American–White mixed, and Native American–Black–White mixed), and Other (including Black-White mixed and Other multiple races).

2. Adjustment of COVID-19 Severity Definitions

To redefine COVID-19 severity in the EPIC-HR trial, we used the definitions from the MOVe-OUT trial (mild COVID-19, moderate COVID-19), as shown as follows:[3,4]

Table S5. COVID-19 Disease Severity Definitions of MOVe-OUT[4]

Disease Severity	Definition
Mild COVID-19	Laboratory-confirmed SARS-CoV-2 infection and ≥1 of the following signs or symptoms: fever >38ºC, chills, cough, sore throat, shortness of breath or difficulty breathing with exertion, fatigue, nasal congestion, runny nose, headache, muscle or body aches, nausea, vomiting, diarrhea, loss of taste, loss of smell<br>And both of the following:<br>• Respiratory rate <20 breaths/min<br>• Heart rate <90 beats/min<br>And either of the following:<br>• SpO2 >93% on room air<br>• Receipt of supplemental oxygen for a reason other than COVID-19, with no increase since the onset of COVID-19 signs or symptoms<br>And both of the following:<br>• No shortness of breath at rest or with exertion<br>• No respiratory failure, shock, or multiorgan dysfunction or failure
Moderate COVID-19	Laboratory-confirmed SARS-CoV-2 infection and ≥1 of the following signs or symptoms: fever >38ºC, chills, cough, sore throat, shortness of breath or difficulty breathing with exertion, fatigue, nasal congestion, runny nose, headache, muscle or body aches, nausea, vomiting, diarrhea, loss of taste, loss of smell<br>And ≥1 of the following:<br>• Respiratory rate ≥20 to <30 breaths/min<br>• Heart rate ≥90 to <125 beats/min<br>• Shortness of breath with exertion<br>And any of the following:<br>• SpO_2 >93% on room air<br>• Receipt of supplemental oxygen for a reason other than COVID-19, with no increase since the onset of COVID-19 signs or symptoms<br>• Receipt of ≤4 L of supplemental oxygen per minute for COVID-19, but not previously receiving supplemental oxygen, regardless of SpO_2<br>And both of the following:<br>• No shortness of breath at rest<br>• No respiratory failure, shock, or multiorgan dysfunction or failure

Abbreviations: COVID-19, coronavirus disease 2019; SARS-CoV-2, severe acute respiratory syndrome coronavirus 2; SpO_2, oxygen saturation.

Definition of respiratory failure, shock and multi-organ dysfunction/failure in MOVe-OUT trial[3]

One or more of the following: respiratory failure, shock, or multi-organ dysfunction/failure.

Respiratory failure is defined based on resource utilization requiring at least 1 of the following:

- Endotracheal intubation and mechanical ventilation
- Oxygen delivered by high-flow nasal cannula (heated, humidified, oxygen delivered via reinforced nasal cannula at flow rates >20 L/min with a fraction of delivered oxygen ≥0.5)
- Noninvasive positive pressure ventilation
- Extracorporeal membrane oxygenation
- Clinical diagnosis of respiratory failure (ie, clinical need for 1 of the proceeding therapies, but preceding therapies not able to be administered in setting of resource limitation)

Shock is defined as systolic blood pressure <90 mmHg, diastolic blood pressure <60 mmHg, or requiring vasopressors.

Multi-organ dysfunction/failure is defined as participants who are acutely ill with evidence of either dysfunction or failure, at the discretion of the investigators, of at least 1 of the following organ systems: respiratory, cardiovascular, renal, hematologic, hepatic, and/or central nervous system.

2-1. Variables Directly Applicable According to the Clinical Trial Definitions>

In the EPIC-HR trial, variables directly applicable to the MOVe-OUT trial (respiratory rate, heart rate, arterial oxygen saturation of pulse oximetry (SpO2), shock) used the same definitions.

2-2. Reclassified Variables

Variables that were reclassified to be applicable to the EPIC-HR trial (Signs or symptoms, Shortness of breath, No respiratory failure) were reclassified as follows:

Signs or symptoms: 0 indicates no symptoms, 1 or more indicates the presence of symptoms.

Shortness of breath: 0 indicates no symptom, 1 and 2 indicate shortness of breath with exertion, 3 indicates shortness of breath at rest.

2-3. Substitute Variables

For respiratory failure, since there were no directly applicable variables, oxygen therapy and mechanical ventilation were used as substitutes.

In both trials, there was a high percentage of missing values for COVID-19 variants (EPIC-HR trial: 33.28%, MOVe-OUT trial: 44.32%; **Supplemental Table S6**). Additionally, the distribution of variants differed significantly between the trials.

Table S6. Proportions of COVID-19 Variants on EPIC-HR and MOVe-OUT Trials

COVID-19 Variants	EPIC-HR (mITT1)	MOVe-OUT (mITT)
Gamma, %	0.34	5.97
Delta, %	65.88	32.53
Mu, %	0.11	11.15
Other variants, %	0.39	6.04
Missing, %	33.28	44.32

Abbreviations: COVID-19, coronavirus disease 2019; mITT, modified intention-to-treat.

Therefore, adjusting both groups using the variants is expected to result in a significantly smaller ESS in the EPIC-HR trial after matching. Moreover, previous studies have shown that the hospitalization and mortality rates during the periods when the Alpha and Delta variants were predominant did not differ significantly.[5] This finding applies to the periods of July to December 2021 for the EPIC-HR trial and May to November 2021 for the MOVe-OUT trial, when these variants were prevalent. Based on these findings, COVID-19 variants were not included as matching variables in the primary analysis. The appropriateness of this analysis plan was also supported by clinical experts.

The analysis, including COVID-19 variants as matching variables, was conducted as sensitivity analysis in two ways:

1. Patients with missing variant information were excluded, and the remaining patients with available variant information were adjusted using the variants as matching variables (Sensitivity analysis 1).

2. Patients with missing variant information in the EPIC-HR trial were assumed to have the Delta variant, and the groups were adjusted using the variants as matching variables (Sensitivity analysis 2).

In this analysis, the EPIC-HR trial used IPD, which made it challenging to evenly distribute the variant information among patients. As over 90% of the patients with missing variant information were assumed to have the Delta variant, these patients were considered to have the Delta variant.

For the MOVe-OUT trial, which used AgD, the proportions of variants were evenly distributed among Gamma, Delta, Mu, and Other variants for both sensitivity analyses.

Supplemental Description 2

Both sensitivity analysis 1 and sensitivity analysis 2 demonstrated additional efficacy of nirmatrelvir-ritonavir compared to molnupiravir with a statistically significant difference of -8.51% (95% CI: –11.73 to –5.27) and -6.69% (95% CI: –9.65 to –3.75), respectively. Although the point estimates in these sensitivity analyses showed better results compared to the primary analysis, the Effective Sample Size (ESS) in the entire mITT1 population of the EPIC-HR trial was significantly reduced to 23. This indicates the limitations of using variants as matching variables.

Table S7. Baseline Characteristics in Sensitivity Analysis 1

Baseline Variable	MOVe-OUT						EPIC-HR							*P* Value vs Original Sample[a] in Total	*P* Value vs Matched Sample[a] in Total
	AgD Mol (N = 709)		AgD Placebo (N = 699)		AgD Total (N = 1408)		Original Sample N/R (N = 601)		Original Sample Placebo (N = 584)		Original Sample Total (N = 1185)		Matched Sample Total (ESS = 23)		
	n	%	n	%	n	%	n	%	n	%	n	%	%		
Sex															
Male	330	46.5	355	50.8	685	48.7	296	49.3	296	50.7	592	50.0	48.6	0.533	1.000
Female	379	53.5	344	49.2	723	51.3	305	50.7	288	49.3	593	50.0	51.4		
Age group															
≤60 years	591	83.4	572	81.8	1163	82.6	479	79.7	442	75.7	921	77.7	82.6	0.002	0.999
>60 years	118	16.6	127	18.2	245	17.4	122	20.3	142	24.3	264	22.3	17.4		
Race															
White only	397	56.0	405	58.0	802	57.0	430	71.5	430	73.6	860	72.6	57.0	<.001	1.000
Black only	39	5.5	34	4.9	73	5.2	24	4.0	10	1.7	34	2.9	5.2		
Asian only	25	3.5	22	3.2	47	3.3	63	10.5	62	10.6	125	10.5	3.3		
Native American	178	25.1	172	24.6	350	24.9	79	13.1	78	13.4	157	13.2	24.9		
Other	70	9.9	65	9.3	135	9.6	5	0.8	4	0.7	9	0.8	9.6		
Obesity status (BMI ≥30 kg/m^2)															
Yes	535	75.5	507	72.5	1042	74.0	231	38.4	206	35.3	437	36.9	74.0	<.001	1.000
No	174	24.5	192	27.5	366	26.0	370	61.6	378	64.7	748	63.1	26.0		
Specific comorbidities															
Diabetes mellitus	107	15.1	117	16.7	224	15.9	59	9.8	66	11.3	125	10.5	15.9	<.001	1.000
Cardiovascular disorders[b]	86	12.1	78	11.2	164	11.6	27	4.5	30	5.1	57	4.8	11.6	<.001	1.000
Time from symptom onset															
≤3 days	339	47.8	335	47.9	674	47.9	413	68.7	402	68.8	815	68.8	47.9	<.001	1.000
>3 days	370	52.2	364	52.1	734	52.1	188	31.3	182	31.2	370	31.2	52.1		
Baseline serology status															
Positive	136	20.1	146	21.9	282	21.0	215	35.8	198	33.9	413	34.9	21.0	<.001	0.999
Negative	541	79.9	520	78.1	1061	79.0	386	64.2	386	66.1	772	65.1	79.0		
COVID-19 Variant															
Gamma	37	9.3	47	12.1	84	10.7	3	0.5	3	0.5	6	0.5	10.7	<.001	1.000
Delta	237	59.8	221	57.0	458	58.4	591	98.3	579	99.1	1170	98.7	58.4		
Mu	75	18.9	82	21.1	157	20.0	2	0.3	0	0.0	2	0.2	20.0		
Other	47	11.9	38	9.8	85	10.8	5	0.8	2	0.3	7	0.6	10.8		

Table S7. Baseline Characteristics in Sensitivity Analysis 1

Baseline Variable	MOVe-OUT						EPIC-HR							*P* Value vs Original Sample[a] in Total	*P* Value vs Matched Sample[a] in Total
	AgD Mol (N = 709)		AgD Placebo (N = 699)		AgD Total (N = 1408)		Original Sample N/R (N = 601)		Original Sample Placebo (N = 584)		Original Sample Total (N = 1185)		Matched Sample Total (ESS = 23)		
	n	%	n	%	n	%	n	%	n	%	n	%	%		
Baseline COVID-19 severity															
Mild	395	55.9	376	53.9	771	55.0	211	35.1	197	33.7	408	34.4	55.0	<.001	1.000
Moderate	311	44.1	321	46.1	632	45.0	390	64.9	387	66.3	777	65.6	45.0		

Abbreviations: AgD, aggregated data; COVID-19, coronavirus disease 2019; ESS, effective sample size; MOL, molnupiravir; N/R, nirmatrelvir-ritonavir.
[a]*P* values were estimated based on χ^2 test for categorical variables.
[b]Refers to serious heart condition (heart failure, coronary artery disease, or cardiomyopathies) in MOVe-OUT AgD.

Figure S10. Distribution of Weights for EPIC-HR Data in Sensitivity Analysis 1

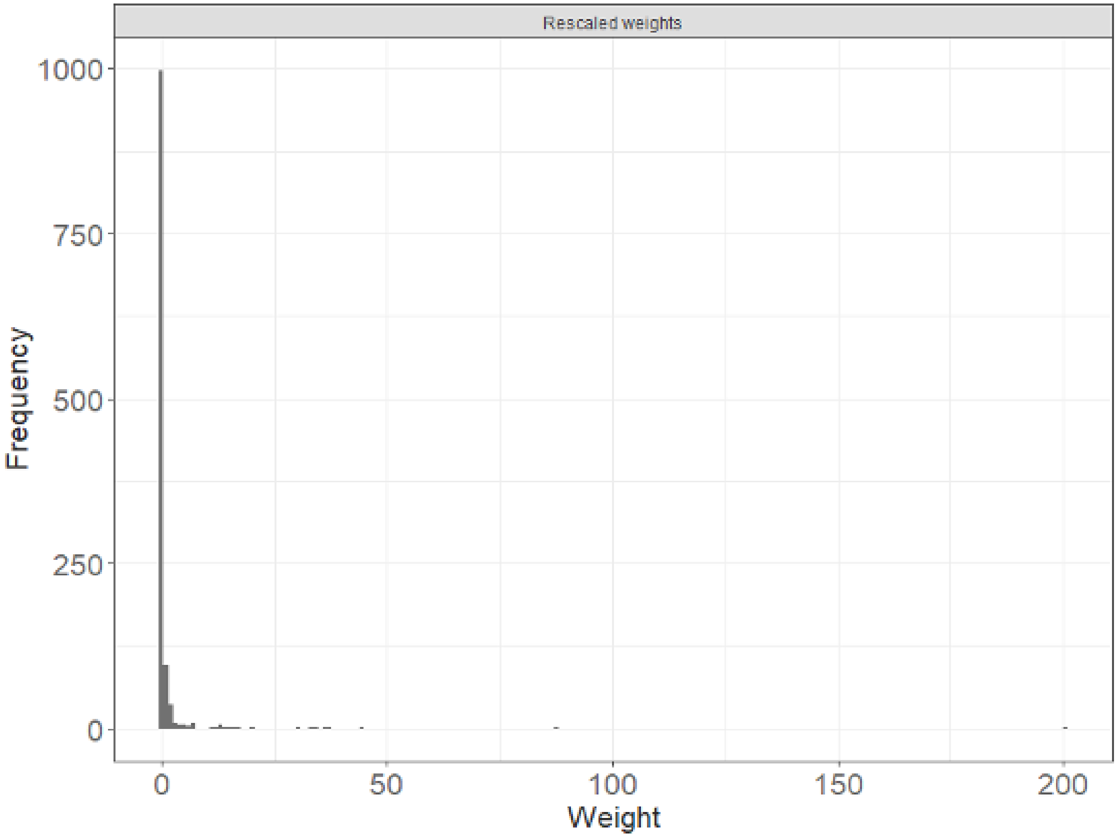

Table S8. COVID-19-Related Hospitalization or All-Cause Death Before/After Matching in Sensitivity Analysis 1

	Intervention			Placebo			Total ESS	N/R: Placebo (A)	Mol: Placebo (B)	A – B	Lower 95% CI	Upper 95% CI
	Total	Outcome		Total	Outcome							
	N	n	%	N	n	%	N					
MOVe-OUT AgD	709	45	6.3	699	64	9.2						
EPIC HR before matching	601	7	1.2	584	56	9.6						
EPIC HR after matching			0.7			12.0	23	-11.3%	-2.8%	-8.5%	-11.73%	-5.27%

Abbreviations: AgD, aggregated data, CI, confidence interval, COVID-19, coronavirus disease 2019, ESS, effective sample size, MOL, molnupiravir, N/R, nirmatrelvir-ritonavir.

Table S9. Baseline Characteristics in Sensitivity Analysis 2

Baseline Variable	MOVe-OUT						EPIC-HR							*P* Value vs Original Sample[a] in Total	*P* Value vs Matched Sample[a] in Total
	AgD Mol (N = 709)		AgD Placebo (N = 699)		AgD Total (N = 1408)		Original Sample N/R (N = 601)		Original Sample Placebo (N = 584)		Original Sample Total (N = 1185)		Matched Sample Total (ESS = 23)		
	n	%	n	%	n	%	n	%	n	%	n	%	%		
Sex															
Male	330	46.5	355	50.8	685	48.7	443	50.6	462	51.3	905	51.0	48.7	0.209	1.000
Female	379	53.5	344	49.2	723	51.3	432	49.4	439	48.7	871	49.0	51.3		
Age group															
≤60 years	591	83.4	572	81.8	1163	82.6	725	82.9	717	79.6	1442	81.2	82.6	0.330	1.000
>60 years	118	16.6	127	18.2	245	17.4	150	17.1	184	20.4	334	18.8	17.4		
Race															
White only	397	56.0	405	58.0	802	57.0	599	68.5	640	71.0	1239	69.8	57.0	<.001	1.000
Black only	39	5.5	34	4.9	73	5.2	41	4.7	29	3.2	70	3.9	5.2		
Asian only	25	3.5	22	3.2	47	3.3	134	15.3	132	14.7	266	15.0	3.3		
Native American	178	25.1	172	24.6	350	24.9	94	10.7	92	10.2	186	10.5	24.9		
Other	70	9.9	65	9.3	135	9.6	7	0.8	8	0.9	15	0.8	9.6		
Obesity status (BMI ≥30 kg/m^2)															
Yes	535	75.5	507	72.5	1042	74.0	304	34.7	314	34.9	618	34.8	74.0	<.001	1.000
No	174	24.5	192	27.5	366	26.0	571	65.3	587	65.1	1158	65.2	26.0		
Specific comorbidities															
Diabetes mellitus	107	15.1	117	16.7	224	15.9	94	10.7	97	10.8	191	10.8	15.9	<.001	1.000
Cardiovascular disorders[b]	86	12.1	78	11.2	164	11.6	30	3.4	38	4.2	68	3.8	11.6	<.001	1.000

Table S9. Baseline Characteristics in Sensitivity Analysis 2

Baseline Variable	MOVe-OUT						EPIC-HR							*P* Value vs Original Sample[a] in Total	*P* Value vs Matched Sample[a] in Total
	AgD Mol (N = 709)		AgD Placebo (N = 699)		AgD Total (N = 1408)		Original Sample N/R (N = 601)		Original Sample Placebo (N = 584)		Original Sample Total (N = 1185)		Matched Sample Total (ESS = 23)		
	n	%	n	%	n	%	n	%	n	%	n	%	%		
Time from symptom onset															
≤3 days	339	47.8	335	47.9	674	47.9	601	68.7	589	65.4	1190	67.0	47.9	<.001	1.000
>3 days	370	52.2	364	52.1	734	52.1	274	31.3	312	34.6	586	33.0	52.1		
Baseline serology status															
Positive	136	20.1	146	21.9	282	21.0	446	51.0	450	49.9	896	50.5	21.0	<.001	1.000
Negative	541	79.9	520	78.1	1061	79.0	429	49.0	451	50.1	880	49.5	79.0		
COVID-19 variant															
Gamma	37	9.3	47	12.1	84	10.7	3	0.3	3	0.3	6	0.3	10.7	<.001	1.000
Delta[‡]	237	59.8	221	57.0	458	58.4	865	98.9	896	99.4	1761	99.2	58.4		
Mu	75	18.9	82	21.1	157	20.0	2	0.2	0	0.0	2	0.1	20.0		
Other	47	11.9	38	9.8	85	10.8	5	0.6	2	0.2	7	0.4	10.8		
Baseline COVID-19 severity															
Mild	395	55.9	376	53.9	771	55.0	326	37.3	306	34.0	632	35.6	55.0	<.001	1.000
Moderate	311	44.1	321	46.1	632	45.0	549	62.7	595	66.0	1144	64.4	45.0		

Abbreviations: AgD, aggregated data; COVID-19, coronavirus disease 2019; ESS, effective sample size; MOL, molnupiravir; N/R, nirmatrelvir-ritonavir.
[a]*P* values were estimated based on χ^2 test for categorical variables.
[b]Refers to serious heart condition (heart failure, coronary artery disease, or cardiomyopathies) in MOVe-OUT AgD.

Figure S11. Distribution of Weights for EPIC-HR Data in Sensitivity Analysis 2

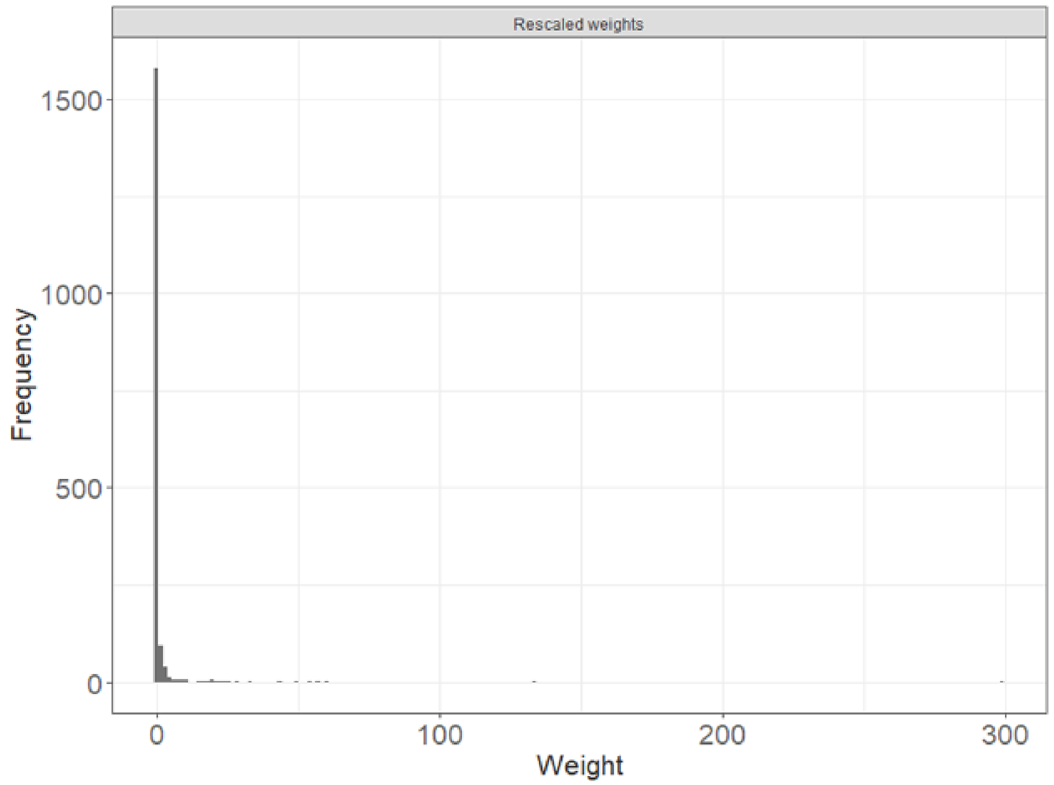

Table S10. COVID-19–Related Hospitalization or All-Cause Death Before/After Matching in Sensitivity Analysis 2

	Intervention			Placebo			Total ESS	N/R - Placebo (A)	Mol - Placebo (B)	A – B	Lower 95% CI	Upper 95% CI
	Total	Outcome		Total	Outcome							
	N	n	%	N	n	%	N					
MOVe-OUT AgD	709	45	6.3	699	64	9.2						
EPIC HR before matching	875	8	0.9	901	60	6.7						
EPIC HR after matching			1.3			10.8	23	-9.5%	-2.8%	-6.7%	-9.65%	-3.75%

Abbreviations: AgD, aggregated data; COVID-19, coronavirus disease 2019; ESS, effective sample size; MOL, molnupiravir; N/R, nirmatrelvir-ritonavir.

References

1. Kamata K, Honda H, Tokuda Y, et al. Post-COVID health-related quality of life and somatic symptoms: a national survey in Japan. *Am J Med Sci*. 2023;366(2):114-123.

2. National Institute for Health and Care Excellence. Multiple Technology Appraisal Therapeutics for people with COVID-19 [ID4038] Committee Papers. Accesseed October 31, 2023. https://www.nice.org.uk/guidance/ta878/evidence/draft-guidance-consultation-committee-papers-pdf-11431471645

3. Jayk Bernal A, Gomes da Silva MM, Musungaie DB, et al. Molnupiravir for oral treatment of Covid-19 in nonhospitalized patients. *N Engl J Med*. 2022;386(6):509-520.

4. Johnson MG, Puenpatom A, Moncada PA, et al. Effect of molnupiravir on biomarkers, respiratory interventions, and medical services in COVID-19: a randomized, placebo-controlled trial. *Ann Intern Med.* 2022;175(8):1126-1134.

5. Esper FP, Adhikari TM, Tu ZJ, et al. Alpha to Omicron: disease severity and clinical outcomes of major SARS-CoV-2 variants. *J Infect Dis.* 2023;227(3):344-352.
